# Clec3b^+^ extraskeletal cells regulate fracture healing and heterotopic ossification

**DOI:** 10.1038/s41413-026-00532-6

**Published:** 2026-07-06

**Authors:** Ezgi Aydin, Jacob A. Moore, Mark Bubnovich, Gabriel Cuilan, Kristin Gebauer, Brenda Kim, Emily Chu, Ugur M. Ayturk

**Affiliations:** 1 Skeletal Health and Orthopedic Research Program, Hospital for Special Surgery, New York, NY USA; 2 Department of Orthopaedic Surgery, Weill Cornell Medicine, New York, NY USA

**Keywords:** Bone, Pathogenesis

## Abstract

Bone healing is orchestrated by multiple cell populations, including those that normally do not produce bone but acquire osteoprogenitor capabilities following injury. Muscle-resident fibroadipogenic progenitors (FAPs) and superficial periosteal cells are among such cells, however their physiologic significance in bone healing and the mechanisms regulating their differentiation are unclear. Here, using the new tamoxifen-inducible *Clec3b.CreERT2* allele, we show that both of these populations can be traced and manipulated in a mouse model. Clec3b^+^ cells are completely absent in marrow and bone-lining surfaces and can differentiate to multiple cell types including osteoblasts during fracture repair and BMP2-induced heterotopic ossification. Orthotopic transplantation assays indicate that Clec3b^+^ FAPs rather than Clec3b^+^ periosteal cells are mobilized to become osteoblasts in these conditions. Further, Clec3b^+^ cells appear functionally distinct from periosteal skeletal progenitors as they exhibit remarkably low chondrogenesis during fracture healing; however, they can form cartilage during heterotopic ossification of muscle and ex vivo culture. Inhibition of WNT-signaling or depletion of Clec3b^+^ cells reduce mineralization during both processes. These data show that extra-skeletal cells that normally do not produce bone, FAPs in particular, are recruited to help repair bone fractures, and could represent a novel target for therapies aimed to enhance fracture healing.

## Introduction

The musculoskeletal system is home to numerous cell populations, including bone-forming osteoblasts, cartilage-forming chondrocytes, and multipotent stem cells.^1–6^ The exact functions and lineage hierarchy of these cells, progenitor populations in particular, remain incompletely described.^7–10^ Yet, various proteins have been proposed as markers of stem and progenitor cells and allow the characterization of functionally distinct populations. The expression of many of these markers localize to the periosteum (such as Ctsk,^3^ Gli1^11,12^ or Acta2/aSMA^13^), an interfacial connective tissue between muscle and bone that is critical for fracture repair.^14–16^ Through prospective functional analyses, mouse cells that co-express *Pdgfra*/CD140A and *Ly6a*/Sca1 (also known as PαS cells) were previously found to possess strong osteogenic potential when cultured in vitro or transplanted into other mice.^17^ The recent emergence of single-cell RNA sequencing technologies led to the detection of Pdgfra^+^ and Ly6a^+^ cells in various tissues, including bone, muscle, fat, and connective tissue.^18–22^ However, to our knowledge, few studies have been reported on the in vivo fate or function of these presumed progenitors in bone with sufficient specificity, as *Pdgfra* is broadly expressed by various mesenchymal-lineage cells,^23,24^ including osteoblasts and osteocytes, whereas *Ly6a* is expressed by a subset of endothelial cells and myeloid cells in bone marrow, making it difficult to track and manipulate Pdgfra^+^ and Ly6a^+^ cells without off-target effects. To address this problem, we identified *Clec3b* (which encodes the secreted protein tetranectin) as a unique marker for Pdgfra^+^ and Ly6a^+^ cells, and engineered an inducible *Clec3b*^*creERT2*^ allele. Here, through lineage-tracing experiments, we show that Clec3b^+^ cells comprise primarily of fibroadipogenic progenitors in skeletal muscle and superficial periosteal cells in the musculoskeletal system. These cells are osteogenically quiescent at baseline but can acquire multi-lineage differentiation capabilities upon injury. *Clec3b*-lineage osteoprogenitors play important roles during both fracture healing and heterotopic ossification, and therefore represent a new and attractive target in the treatment of traumatic musculoskeletal injuries.

## Results

### Clec3b expression marks osteogenically inactive extra-skeletal cells

To perform in vivo tracing experiments, we searched for marker transcripts uniquely expressed by PDGFRA^+^ SCA1^+^ cells. We evaluated single-cell RNA-seq data generated by our group^25^ and others.^18,20,26^ By analyzing *Tmem100*-lineage cells (that include muscle, periosteal and bone marrow stromal cells) obtained from whole mouse lumbar spines of *Tmem100*^*creERT2/+*^*; R26*^*Ai14.tdTomato*^ mice (3 days after tamoxifen pulse at P10, Fig. S1a–c), we found that *Clec3b* expression is a marker for Pdgfra^+^ Ly6a/Sca1^+^ cells (Fig. S1d). Retrospective analysis of published single cell RNA-seq data^18,20,26^ also show a high degree of overlap between Clec3b^+^ cells and Pdgfra^+^ Ly6a/Sca1^+^ cells in muscle, fat and connective tissues (Fig. S1e). Previous reports indicate the importance of *Clec3b-*encoded tetranectin in the healing of bone, connective tissue and skin injuries.^27–29^ We therefore hypothesized that *Clec3b*-expression marks PDGFRA^+^ SCA1^+^ cells that contribute to bone formation by differentiating to osteoblasts in vivo.

**Fig. 1:**
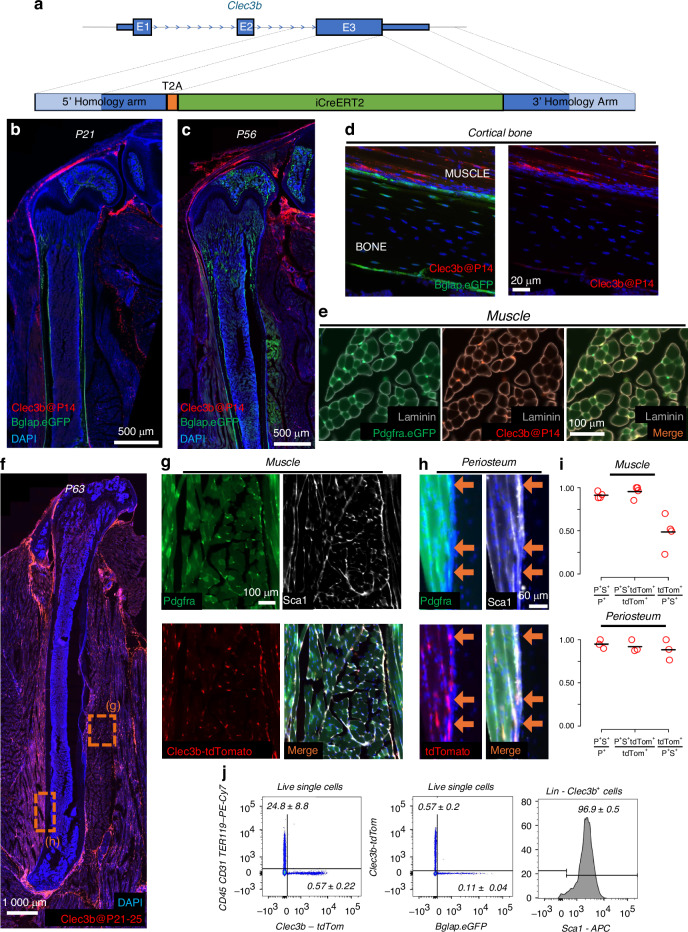
*Clec3b*.creERT2 allele is expressed by muscle, outer periosteum and connective tissue cells upon tamoxifen treatment. **a** A T2A-iCreERT2 construct was knocked in at the 3′ end of the Clec3b protein coding region with CRISPR-Cas9. **b** *Clec3b*^*creERT2*^*; R26*^*Ai14v.tdTomato*^; *Bglap*^*eGFP*^ mice were treated with a single tamoxifen injection at P14. One week later, tdTomato^+^ cells were detected in muscle, superficial periosteum and connective tissue. **c** Six weeks after tamoxifen treatment, tdTomato^+^ cells were found in the same locations (*n* = 3). **d** High magnification imaging of diaphysis from (**c**) confirms that *Clec3b*-lineage cells do not overlap with Bglap.eGFP^+^ osteoblasts. **e** High magnification imaging shows Clec3b-lineage cells in laminin-labeled interstitial muscle overlapping with Pdgfra.eGFP^+^ cells. **f** Six weeks after tamoxifen treatment at weaning, no tdTomato^+^ cells are found in bone tissue. **g** High magnification imaging of muscle depicts tdTomato^+^ cells stained with Pdgfra- and Sca1-recognizing antibodies. **h** High magnification imaging depicting Pdgfra^+^ Sca1^+^ tdTomato^+^ cells (arrows) in the outer periosteum. **i** Quantification of the overlap between Pdgfra, Sca1, and tdTomato expression depicted in (**g**, **h**) (P Pdgfra, S Sca1, *n* = 3-4 mice). **j** Flow cytometry analysis indicates that Clec3b-lineage cells do not overlap with leukocytes, endothelial cells, red blood cells or osteoblasts, and almost all Clec3b^+^ cells are also Sca1^+^ (Data are presented as mean + standard deviation, *n* = 3 samples)

To identify and study *Clec3b*-lineage cells in vivo, we generated a *Clec3b.creERT2* knock-in allele using CRISPR-Cas9 (Fig. 1a and Fig. S2, see “Methods”). We confirmed that the *Clec3b.creERT2* allele did not cause any gross anomalies in mice, and specifically did not alter cortical or trabecular bone properties (Fig. S2b, c). We bred male *Clec3b*^*creERT2/+*^ mice with Ai14.R26.tdTomato female mice, and treated their *Clec3b*^*creERT2/+*^*; R26*^*tdTomato/+*^ progeny with tamoxifen, in order to permanently activate tdTomato-expression in Clec3b^+^ cells. Following a single tamoxifen pulse at P14, we evaluated the fate of *Clec3b*-expressing cells at multiple tissues in the musculoskeletal system. One week later, we observed tdTomato^+^ cells predominantly in skeletal muscle and connective tissues (Fig. 1b). Interestingly, we did not detect any tdTomato^+^ cells in the marrow, endosteum or growth plates of long bones or spine (Fig. 1b, c and Fig. S3a, b); however, some tdTomato^+^ cells were present in the superficial periosteum (Fig. 1d). This was also true for calvaria, where tdTomato^+^ cells were absent in the sutures, dura mater and marrow, but were present in the outer periosteum (Fig. S3c). We did not observe a meaningful change in this anatomic distribution during a 4 month-chase following tamoxifen treatment (Fig. S3d, e): *Clec3b*-lineage cells did not migrate to the bone lining layer or inside the mineralized matrix, or overlap with Bglap.eGFP^+^ osteoblasts, indicating that they do not undergo osteogenic differentiation during postnatal growth of the skeleton (Fig. 1d and Fig. S4).

In connective tissues, tdTomato^+^ cells were localized to the peripheral layers (Fig. S3a). In skeletal muscle, *Clec3b*-lineage cells were attached to muscle fibers, and expressed the *Pdgfra.eGFP* reporter allele, suggesting that they represent a population of fibroadipogenic progenitors (FAPs, Fig. 1e). Consistent with this, six weeks after 3x tamoxifen treatment at P21, P23 and P25, all tdTomato^+^ cells in skeletal muscle and periosteum stained positive for PDGFRA and SCA1 (Fig. 1f–i), and flow cytometry showed that almost all Clec3b^+^ cells are SCA1^+^ (Fig. 1j).

As we found that approximately half of all Pdgfra^+^ Sca1^+^ fibroadipogenic progenitors were tdTomato^+^ in skeletal muscle (Fig. 1i), we next tested whether this partial overlap is due to inefficient recombination of the Cre-allele, or if *Clec3b* is only expressed by a subset of FAPs. Analysis of single cell RNA-seq data obtained from mouse skeletal muscle by 3 other groups revealed that virtually all Pdgfra^+^ and Ly6a/Sca1^+^ cells also express the *Clec3b* transcript (>96% overlap, Fig. S5). Further, RNAscope analysis showed that Pdgfra^+^ cells in mouse skeletal muscle are also Clec3b^+^, and vice versa (>90% overlap, Fig. S6). These data indicate that *Clec3b*-expression marks almost all FAPs in muscle, in addition to cells on the outer layers of periosteum and connective tissues, whereas it is absent in multiple anatomic locations traditionally associated with stem/progenitor cell activity such as the growth plate, marrow and endosteum. Importantly, we did not observe a meaningful number of tdTomato^+^ cells in the absence of tamoxifen (at baseline or after injury), indicating that the *Clec3b.creERT2* allele is not leaky (Fig. S3f).

### Clec3b-lineage cells are mobilized to assist with bone healing

**Fig. 2:**
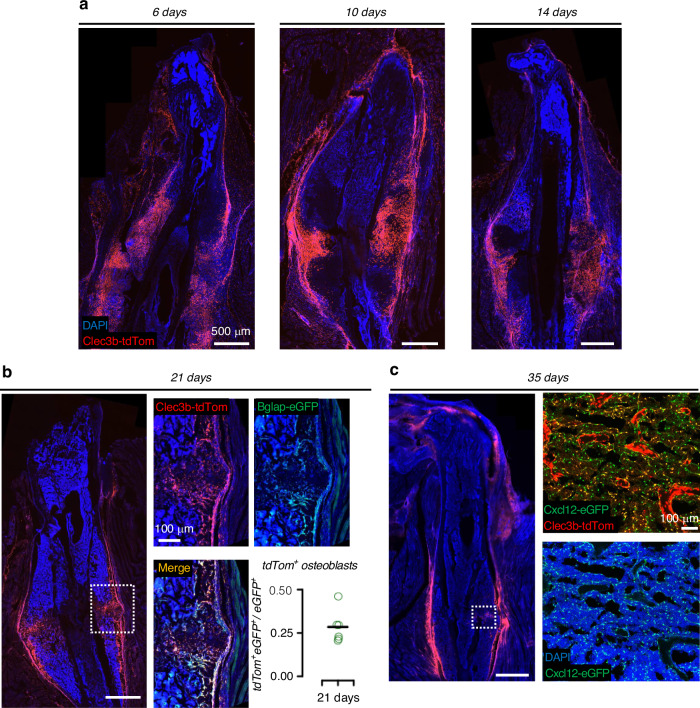
Bone injury induces osteogenic differentiation of Clec3b^+^ cells. *Clec3b*-lineage reporter mice were treated with tamoxifen between 3 and 4 weeks and subjected to stabilized femoral diaphyseal fracture surgeries at 6 weeks. **a** Fluorescent images show Clec3b-lineage cell accumulation in the fracture callus over the 5 weeks post-fracture (*n* = 3–5 mice/timepoint). **b** At 21-days, many tdTomato^+^ cells localize to the newly formed cortical shell at the periphery of the callus. On average, 28% of all Bglap.eGFP^+^ osteoblasts are also tdTomato^+^ (*n* = 5 mice). **c** High magnification imaging depicts the overlap between tdTomato^+^ cells and Cxcl12.eGFP^+^ bone marrow stromal cells in the regenerating marrow at 35 days

Having found that Clec3b^+^ cells are osteogenically inactive during postnatal skeletal growth, we next asked whether they can be stimulated by injury. We confirmed that Clec3b^+^ cells are osteogenically activated in response to multiple forms of bone damage, including monocortical drill hole and unstabilized/stabilized long bone fractures (Fig. S7 and Fig. 2a). To understand how Clec3b^+^ cells might be mobilized to migrate to the fracture site and participate in healing, and which specific cell types they differentiate to, we examined *Clec3b*^*creERT2/+*^*; R26*^*tdTomato/+*^ femora over 35 days post-fracture surgery (Fig. 2). At the 6- and 10-day time-points, the majority of *Clec3b*-lineage tdTomato^+^ cells localized to the fibrotic mid-callus, while few tdTomato^+^ cells were found in the temporal, avascular cartilage tissue.

At 21-days, we confirmed osteogenic differentiation of tdTomato^+^ cells based on *Bglap.eGFP* expression, and found that at least ~28% of callus osteoblasts on average were of *Clec3b*-lineage, which could potentially be higher after accounting for inefficiency of tamoxifen-mediated recombination (Fig. 2b). These data show that although Clec3b^+^ cells appear quiescent at baseline, they are capable of osteogenic differentiation to contribute to bone healing. Interestingly, *Clec3b*-lineage cells also contribute to the reconstruction of bone marrow stroma (similar to Gli1^+^ periosteal cells as previously reported^11^): Following fracture surgeries, we observed tdTomato^+^ cells with a reticular morphology inside the marrow cavity (Fig. 2c). These cells overlapped with Cxcl12.eGFP^+^ cells that appeared to be positioned around vasculature, indicating their conversion to bone marrow stromal cells.

Our analysis of uninjured musculoskeletal tissues had indicated that *Clec3b*-expression is limited to Pdgfra^+^ and Sca1^+^ cells, primarily FAPs in skeletal muscle. To determine whether *Clec3b*-expression is maintained in differentiating FAPs or induced in other osteoprogenitors that participate in callus formation, we treated *Clec3b*-lineage reporter mice with tamoxifen after fracture surgery. However, neither 1-week nor 2-week post-fracture treatment resulted in the labeling of any cells within the fracture callus at 3 weeks (Fig. S8a, b); tdTomato^+^ cells were rather limited to the muscle tissue surrounding the callus. We also purified tdTomato^+^ cells from fracture calluses 1-week post-surgery (with tamoxifen injections performed prior to surgery) and performed qRT-PCR, which identified a significant depletion of *Clec3b*-expression compared to tdTomato^+^ cells obtained from intact muscle (Fig. S8c). These data indicate that *Clec3b*-expression is downregulated shortly after bone injury (presumably as part of differentiation into myofibroblasts and ultimately osteoblasts) and remains limited to Pdgfra^+^ Sca1^+^ cells surrounding bone tissue.

As we detected Clec3b^+^ cells in both the superficial periosteum and muscle fascia, we next wanted to distinguish the relative contributions of these different populations to bone repair and determine whether they overlap with other progenitor populations previously implicated in fracture healing and heterotopic ossification. Recent findings by other groups suggest that muscle-based Clec3b^+^ cells play a more important role compared to their periosteal counterparts in response to injury: Mo et al. showed that the Sca1^+^ subpopulation of Lepr^+^ cells in the periosteum (which also express *Clec3b*) exhibits poor osteogenic and chondrogenic capabilities in vitro.^30^ Cao et al. also reported that Sca1^+^ periosteal cells have limited ability to expand and differentiate in an engraftment model.^31^ On the other hand, using a transplantation approach, Julien et al. showed that muscle-resident Prrx1-lineage stromal cells contribute to the healing of long bone fractures, by undergoing chondrogenic differentiation.^26^ Our analysis of single cell RNA-seq data derived from both unfractionated and *Prrx1*-lineage skeletal muscle cells had confirmed that *Clec3b* is an excellent marker for Pdgfra^+^ and Ly6a/Sca1^+^ fibroadipogenic progenitors (Fig. S5). A similar analysis of previously published datasets indicated that *Clec3b* labels a small fraction of Pdgfra^+^ Ly6a/Sca1^+^ cells in the periosteum (Fig. S9). Thus, Clec3b^+^ cells overlap specifically with muscle fibroadipogenic progenitors previously identified with other marker proteins (such as *Tie2*,^32^ in addition to Pdgfra^+^ and Ly6a/Sca1^+^). Importantly, many of these markers are also expressed by mesenchymal progenitors or cells already committed to osteogenesis in marrow, growth plate, and endosteum (such as *Prrx1* and *Pdgfra*^24^), while others also label myogenic progenitors (such as *Gli1*^33^ and *Hoxa11*^34^). Clec3b^+^ tendon cells exhibit a similar transcriptome in datasets obtained from *Vegfc*- and *Hoxa11*-lineage cells^22,35^ (Fig. S9), which were previously implicated as progenitors for heterotopic ossification of connective tissue.

**Fig. 3:**
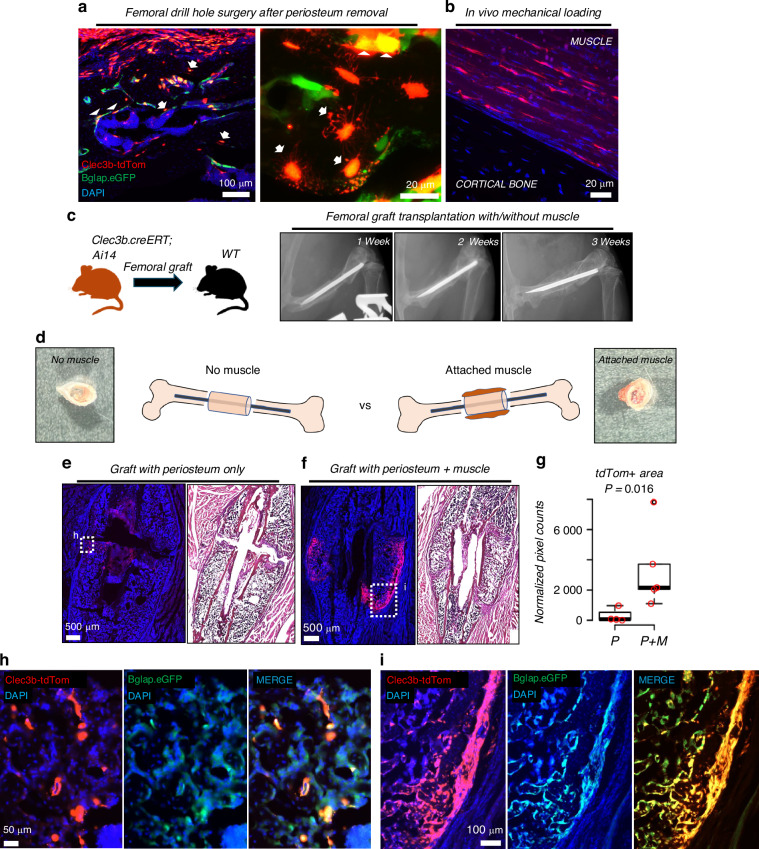
Muscle-resident Clec3b^+^ cells play a bigger role in bone healing than periosteal Clec3b^+^ cells. **a** Femoral drill hole injury, following removal of periosteum, induces migration of *Clec3b*-lineage cells into the healing bone tissue, giving rise to tdTomato^+^ and Bglap.eGFP^+^ osteoblasts (arrowheads) and osteocytes (arrows) 3 weeks later (*n* = 3). **b** No tdTomato^+^ osteocytes are found in tibial diaphysis following 2 weeks of in vivo mechanical loading (*n* = 4). **c** Femoral grafts with or without surrounding muscle tissue are obtained from Clec3b-lineage reporter mice and transplanted into femoral defects created in littermates that lack tdTomato expression. X-ray images depict the integration of grafts through endochondral bone formation. **d** Photographs depict bone grafts with and without muscle. **e** Femoral grafts that do not contain surrounding muscle tissue integrate with the contribution of few tdTomato^+^ cells to callus formation (*n* = 4). **f** On the other hand, abundant tdTomato^+^ cells are found in calluses formed by muscle-containing grafts (*n* = 5). **g** A significantly higher amount of tdTomato^+^ cells is found in calluses formed by muscle-containing grafts. (Wilcoxon rank sum exact test, *n* = 4, 5). **i**, **j** High magnification images of tdTomato^+^ cells depict osteogenic differentiation, indicated by *Bglap.eGFP*-expression

To further interrogate the potential contribution of periosteal *Clec3b*-lineage cells to bone healing, we performed multiple experiments: First, we surgically removed the periosteum and then induced drill-hole injuries on the femoral diaphysis. Three weeks later, we found that *Clec3b*-lineage cells had become Bglap.GFP^+^ osteoblasts, osteocytes and bone marrow stromal cells, indicating that muscle-based Clec3b^+^ cells are osteogenically activated by bone injury (Fig. 3a). Second, we performed in vivo mechanical loading on the tibia for 2 weeks, to determine whether Clec3b^+^ periosteal cells would respond to osteoanabolic stimulation in the absence of injury. However, we were unable to identify any tdTomato+ osteocytes throughout the tibial cortex (Fig. 3b), indicating that Clec3b^+^ cells did not undergo osteogenic differentiation. Third, we harvested diaphyseal bone grafts with or without surrounding muscle tissue from *Clec3b*^*creERT2/+*^*; R26*^*tdTom/+*^ mice, and grafted them into surgically created femoral defects in littermate mice that lack tdTomato-expression (Fig. 3c, d). These defects healed through endochondral ossification with the contribution of graft cells to callus tissue. Three weeks after surgery, we observed some tdTomato^+^ cell presence in fracture calluses treated with grafts that did not contain muscle (Fig. 3e), whereas tdTomato^+^ cells were significantly more abundant in calluses treated with muscle-containing grafts (Fig. 3f, g and Fig. S10). In both cases, we confirmed that tdTomato+ cells gave rise to Bglap.eGFP^+^ osteoblasts and osteocytes (Fig. 3h, i). Altogether, these data show that bone injury induces osteogenic differentiation of Clec3b^+^ cells, and the majority of these cells are likely recruited from skeletal muscle rather than the outer periosteum.

### Clec3b-lineage single cell transcriptome during fracture healing vs homeostasis

**Fig. 4:**
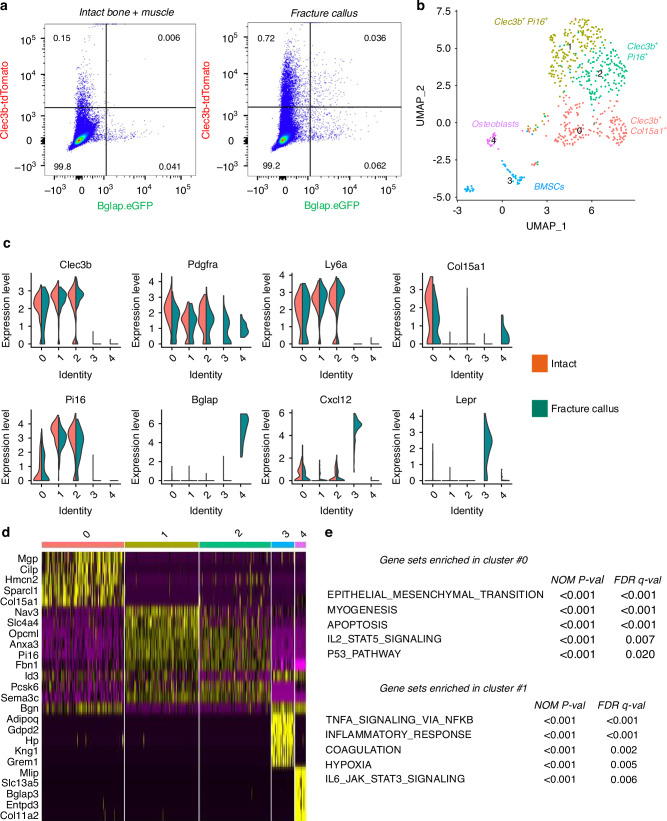
Single-cell RNA-seq reveals subsets and progeny of Clec3b^+^ cells during fracture healing. **a** Flow cytometry analysis depicts lack of overlap between *Clec3b*-lineage tdTomato^+^ cells and Bglap.eGFP^+^ osteoblasts in intact bone and muscle specimens, whereas tdTomato^+^ eGFP^+^ cells are found in fracture calluses. **b** UMAP plot depicts 5 transcriptionally distinct clusters of *Clec3b*-lineage cells obtained from intact and fractured femoral specimens. **c** Violin plots show that Clec3b^+^ cells are found in both intact and fracture specimens. *Pi16* and *Col15a1* expression distinguishes subsets of Clec3b^+^ cells in both groups. Osteoblasts (Bglap^+^, #4) and BMSCs (Lepr^+^, #3) are found in the fracture callus but not intact specimens. **d** Heatmap depicts the similarity of cluster #1 and #2 transcriptomes, while the other clusters exhibit distinct gene expression profiles. **e** Gene set enrichment analysis indicates a significant enrichment for cellular differentiation, muscle development, and cell death-associated transcripts in cluster #0, whereas the cluster #1 transcriptome is enriched for inflammation-associated transcripts

We next aimed to determine the transcriptional diversity of Clec3b^+^ cells and how this diversity changes during fracture healing. We obtained tdTomato^+^ cells from intact femora (and surrounding muscle) or fracture calluses 3 weeks after surgery, and performed single cell RNA-seq (Fig. 4a). Our initial analysis demonstrated the presence of various hematopoietic and endothelial populations among *Clec3b*-lineage cells; however, these populations did not express meaningful levels of tdTomato, indicating that they were flow cytometry artifacts (Fig. S11). Thus, after the exclusion of these non-mesenchymal cell clusters (including endothelial cells), we detected 5 distinct clusters collectively from intact bone & muscle and fracture callus specimens (Fig. 4b). Clusters #0, #1 and #2 expressed high levels of Clec3b^+^ and were found in both sets of specimens. We found that these cells can be distinguished by the “universal fibroblast” markers previously identified by Buechler et al.,^36^ such that cells in cluster #0 express *Col15a1*, whereas *Pi16* expression is specific to those in #1 and #2 (Fig. 4c, d). The presence of these subsets among Clec3b^+^ cells is consistent with muscle and connective tissue single-cell RNA-seq data published by other investigators (Figs. S5  and S9). Gene set enrichment analysis confirmed a clear distinction between the transcriptomes of these clusters, as cluster #0 expressed genes associated with adipogenesis, myogenesis, and collagenous extracellular matrix deposition (Fig. 4e). On the other hand, cells in clusters #1 and #2 were enriched for transcripts associated with inflammatory signaling events, including TNFα and IL6-JAK-STAT pathways. Importantly, we did not detect meaningful expression of transcripts associated with proliferation in any of the clusters (Fig. S12), and no cells exhibited an expression profile consistent with the description of skeletal stem cells by Chan et al.^1^ (i.e., Cd200^+^Cd105/Eng^-^ or Cd200^-^ Cd105/Eng^-^). Clusters #3 and #4 solely comprised of cells originating from the fracture callus. Consistent with histology findings, cells in cluster #3 expressed markers of bone marrow stromal cells (such as *Lepr*, *Cxcl12*, *Adipoq, Kitl*), whereas cells in cluster #4 represented osteoblasts, as shown by the expression of *Bglap*, *Ifitm5*, and *Dmp1* (Fig. 4b, c). These data show the presence of Clec3b^+^ cell subsets and the emergence of osteoblast-lineage cells following bone fracture, consistent with our histology results.

### Clec3b-lineage cells participate in heterotopic ossification of skeletal muscle and the ankle joint

**Fig. 5:**
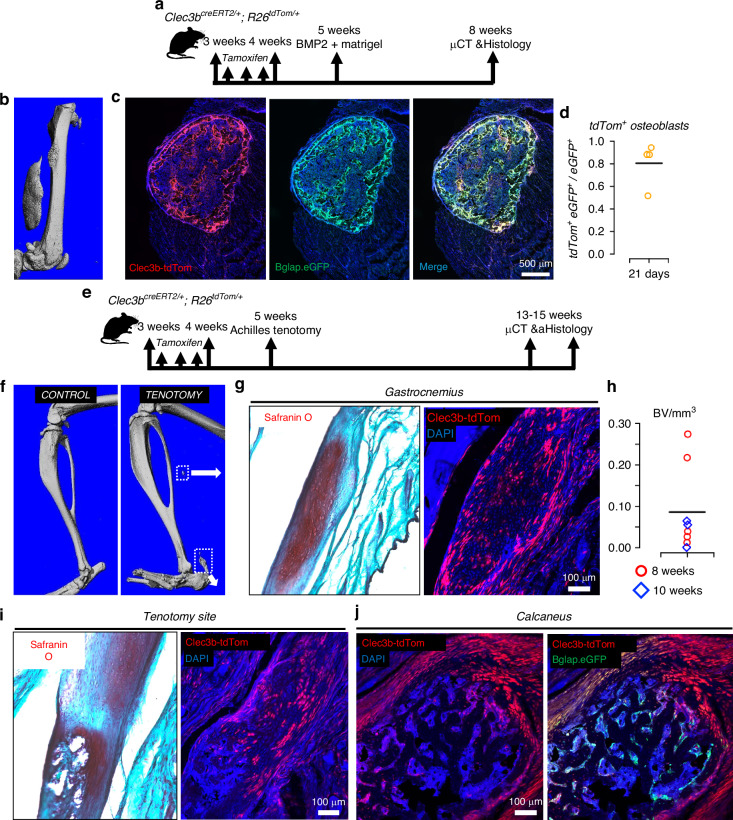
*Clec3b*-lineage cells participate in heterotopic ossification of skeletal muscle and Achilles tendon. **a** *Clec3b*-lineage reporter mouse was treated with tamoxifen between 3 and 4 weeks. Intramuscular delivery of rhBMP2 mixed with a matrigel scaffold was performed at 5 weeks. **b** Three weeks after rhBMP2 injection, robust ectopic bone formation was observed adjacent to the right femur, as depicted in 3D reconstruction of a representative specimen. **c** Fluorescent images of ectopic bone. *Clec3b*-lineage cells are abundantly present in HO, overlapping with Bglap.eGFP^+^ osteoblasts. **d** The majority of osteoblasts in BMP2-induced ectopic bones are of *Clec3b*-lineage, as indicated by the overlap between tdTomato and *Bglap.eGFP* fluorescence. **e** Clec3b-lineage reporter mice were treated with tamoxifen between 3 and 4 weeks. Unilateral Achilles tenotomy was performed 1 week later, and mice were sacrificed 8–10 weeks post-surgery. **f** 3D reconstruction of μCT data from uninjured (left) and injured (right) limbs (*n* = 8 mice). The calcaneus was malformed, and ectopic mineralization was observed in the gastrocnemius muscle and ankle joint of all mice. **g** Safranin O-staining and fluorescent imaging of adjacent sections depict chondrogenic differentiation of tdTomato^+^ cells. **h** Quantification of total ectopic calcification in the gastrocnemius muscle and ankle joint (excluding the calcaneus). We did not observe meaningful differences between the 8-week (*n* = 5) and 10-week (*n* = 3) timepoints. **i** Safranin-O-stained and fluorescent images depict tdTomato^+^ cells, including chondrocytes at the tenotomy site. **j** Fluorescent images of the calcaneus depict abundant tdTomato^+^ cells, some of which overlap with Bglap.eGFP^+^ osteoblasts

As our data indicate that muscle-based Clec3b^+^ cells convert to osteoprogenitors during fracture healing, we next asked whether these cells also contribute to pathologic muscle mineralization, i.e., heterotopic ossification (HO). We therefore induced heterotopic ossification by injecting recombinant human BMP2 into the right caudal thigh muscle (Fig. 5a). Three weeks later, we observed large ectopic bone nodules adjacent to the femoral diaphysis (Fig. 5b), and these mineralized structures were densely populated with *Clec3b*-lineage cells (Fig. 5c, d), many of which had become osteoblasts and osteocytes.

Having confirmed that Clec3b^+^ FAPs can form bone in muscle, we next tested whether Clec3b^+^ cells also contribute to traumatic heterotopic ossification induced by connective tissue injuries. We did this by severing the right Achilles tendon in mice, and confirmed ectopic calcification around the calcaneus and the gastrocnemius muscle at 8–10 weeks following injury (Fig. 5e, f). Histologic analysis confirmed that tdTomato^+^ cells differentiate to chondrocytes, Bglap.eGFP^+^ osteoblasts, and osteocytes following injury (Fig. 5g–j), indicating the contribution of Clec3b^+^ cells to heterotopic ossification in this model.

### Clec3b^+^ cell depletion alters fracture healing and heterotopic ossification

**Fig. 6:**
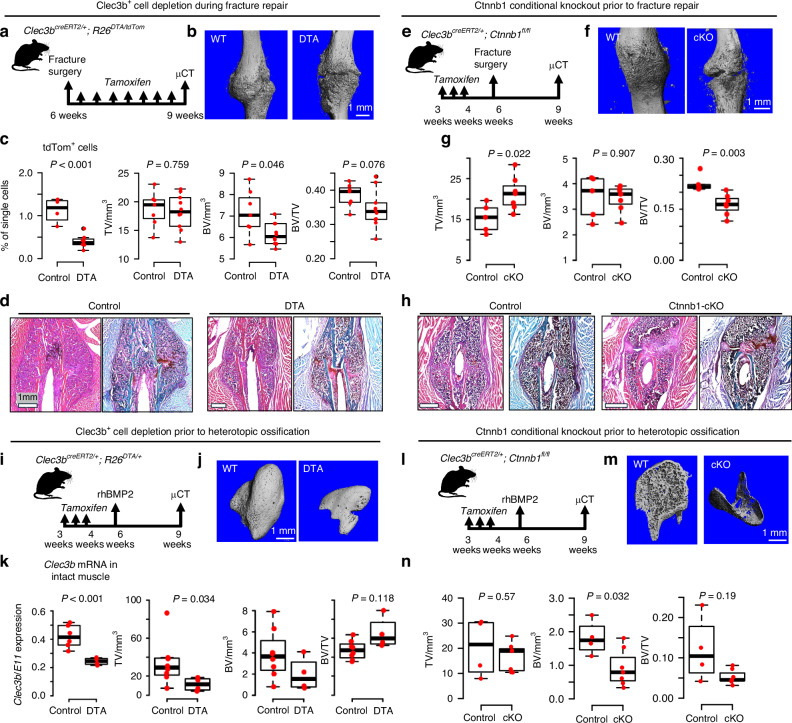
Manipulation of Clec3b^+^ cells alters fracture healing and heterotopic ossification. **a** Clec3b^+^ cells were ablated by activation of a diphtheria toxin (DTA) expression allele through repeated tamoxifen injections every 3 days for 3 weeks, during fracture healing. **b** 3D reconstruction images of representative fracture calluses from DTA and WT (i.e., *Clec3b*^*+/+*^*; R26*^*DTA*^) mice. **c** A significant reduction in callus bone volume (BV) and trend towards reduction in mineralized fraction (BV/TV) were observed in DTA mice. No changes were found in the total volume (TV) of the callus (Two-tailed unpaired *t*-test, *n* *=* 7–10 female littermate mice/group). Flow cytometry analysis indicated a 65% reduction in *Clec3b*-lineage cells in DTA mice (two-tailed unpaired *t*-test, *n* = 4–8). **d** Representative histology images depicting the fracture callus in both groups. **e** *Ctnnb1* was conditionally deleted in Clec3b^+^ cells through tamoxifen treatment between 3 and 4 weeks. Fracture surgery was performed at 6 weeks, and mice were sacrificed at 9 weeks for analysis. **f** 3D reconstruction images of representative fracture calluses from wild-type (*Clec3b*^*+/+*^*; Ctnnb1*^*fl/+*^, WT) and Ctnnb1-conditional knockout (*Clec3b*^*CreERT2*^*; Ctnnb1*^*fl/fl*^, cKO) mice. **g** We observed a significant increase in TV and reduction in BV/TV in cKO mice (two-tailed unpaired *t*-test, *n* = 5–7 male littermate mice/group). **h** Representative histology images depicting the fracture callus in both groups. **i** Muscle ectopic mineralization was induced in DTA and WT control mice by a single intramuscular rhBMP2 injection at 5 weeks, following tamoxifen treatment between 3 and 4 weeks. **j** 3D reconstructions of representative specimens from WT and DTA groups. **k** A significant reduction in total volume (TV) was found in DTA mice compared to WT controls (two-tailed unpaired *t*-test, *n* = 4–8 male littermate mice/group). **l** Muscle ectopic mineralization was induced in *Ctnnb1*-cKO and WT (*Clec3b*^*+/+*^*; Ctnnb1*^*fl/+*^) groups. **m** 3D reconstructions of representative specimens from WT and cKO groups. **n** A significant reduction in bone volume (BV) was found in conditional knockout mice compared to WT controls (two-tailed unpaired *t*-test, *n* = 4–7 female littermate mice/group)

As Clec3b^+^ cells participate in both fracture healing and heterotopic ossification by becoming osteoblasts, we asked whether disabling them would inhibit bone formation in either context. To answer this question, we generated *Clec3b*^*creERT2/+*^*; R26*^*DTA/+*^ mice, which allowed us to ablate Clec3b^+^ cells through induction of endogenous diphtheria toxin expression upon tamoxifen injection (Fig. 6a). We used this model to kill Clec3b^+^ cells during fracture healing, by treating mice with tamoxifen every 3 days (starting the day before surgery) for 3 weeks. At the end of this period, FACS-based quantification indicated a 65% reduction in tdTomato^+^ cells in the muscle tissue of DTA mice (Fig. 6c). μCT analysis showed a reduction in fracture callus mineralization, as indicated by lower bone volume (BV) and bone volume fraction (BV/TV) compared to littermate control mice (*P* < 0.05); however, we did not detect a change in callus size (Fig. 6c). Similarly, deletion of *Ctnnb1* (encoding β-catenin, the master transcription factor in the WNT-signaling pathway), a known regulator of osteoprogenitor cell activation,^11,37^ prior to fracture surgery resulted in reduced callus bone volume fraction in mice (Fig. 6e–g).

Using the cell ablation approach, we also depleted Clec3b^+^ cells following BMP2-induced heterotopic ossification in muscle. Interestingly, continuous tamoxifen treatment following BMP2-delivery did not lead to a reduction in ectopic bone formation (Fig. S13a, b), but rather suggested a trend towards increased bone mass due to Clec3b^+^ cell depletion (Fig. S13c). Histologic analysis indicated the presence of tdTomato- cells (osteocytes in particular) in mice with DTA alleles (Fig. S13d). This suggested to us that induced apoptosis of *Clec3b*-lineage cells mobilizes surviving tdTomato- cells to differentiate and form bone (resulting in an increase in tdTomato- osteocytes, Fig. S13e), consistent with the reported mineralization-triggering effect of toxins and inflammation previously reported in genetic and BMP2-induced models of heterotopic ossification.^38–41^ To circumvent these potential artifactual changes, and test the effects of Clec3b^+^ cell loss of function, we depleted Clec3b^+^ cells by treating *Clec3b*^*creERT2/+*^*; R26*^*DTA/+*^ mice with tamoxifen prior to BMP2-injection (Fig. 6i). We measured a 42% reduction in *Clec3b*-expression in intact muscle, indicating decreased Clec3b^+^ FAPs (Fig. 6k). We also found that such treatment resulted in reduced volume of ectopic mass (i.e. TV, Fig. 6j, k), confirming our initial hypothesis that Clec3b^+^ cell loss would negatively affect heterotopic ossification. Consistent with this result, deletion of *Ctnnb1* also resulted in a significant reduction in ectopic bone volume compared to littermate control mice (Fig. 6l–n).

Trauma-induced heterotopic ossification is coupled with adipogenesis,^42^ which is recapitulated in animal models,^22,43,44^ including our own (Fig. S14a). As WNT-signaling is a known regulator of cell fate decision between osteogenesis and adipogenesis in the bone marrow,^45^ we next tested whether *Ctnnb1*-deletion in Clec3b^+^ cells promotes adipose tissue accumulation inside ectopic bone tissue. However, immunostaining for perilipin did not show increased adipocyte accumulation (Fig. S14b, c). Altogether, these data indicate that disabling Clec3b^+^ cells (through targeted depletion or blocking WNT-signaling) significantly reduces ectopic mineralization in skeletal muscle.

### Clec3b^+^ cells exhibit context-specific chondrogenic differentiation

**Fig. 7:**
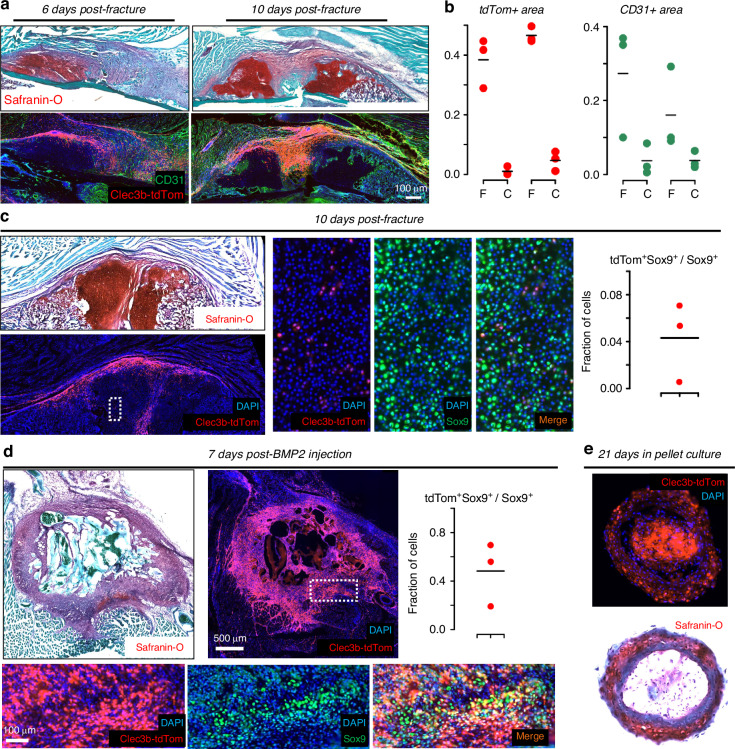
Clec3b^+^ cells undergo chondrogenic differentiation in a context-specific manner. **a** Representative histology images depicting Safranin-O and CD31-staining and fluorescent imaging of the cartilaginous fracture callus 6- and 10-days post-surgery. **b** Quantification of tdTomato^+^ and CD31^+^ cells indicates a significant enrichment for both cell types in the fibrotic callus (F) compared to the cartilaginous callus (C) (*P* < 0.05, *n* = 3 mice/timepoint). **c** Sox9-staining of the cartilaginous callus 10 days after fracture reveals minimal overlap between Clec3b-tdTomato^+^ cells and chondrocytes (*n* = 3 mice). **d** Safranin-O and Sox9-staining of ectopic bones in muscle induced by BMP2-injection depicts Clec3b-tdTomato^+^ cells differentiating into chondrocytes (*n* = 4 mice). **e** Fluorescent and Safranin-O-stained sections of a representative chondrocyte pellet formed ex vivo by primary tdTomato^+^ cells isolated from the hindlimb muscles of *Clec3b*-lineage reporter mice (*n* = 4 samples)

As we detected few *Clec3b*-lineage cells in the cartilaginous fracture callus, we next wanted to test whether Clec3b^+^ cells are incapable of chondrogenic differentiation. We first quantified the contribution of Clec3b^+^ cells to chondrocytes shortly after bone fracture, by examining callus 6- and 10-days post-surgery (Fig. 7a). On average, less than 5% of Safranin-O-stained avascular cartilage contained tdTomato^+^ cells at either timepoint (Fig. 7b). Consistent with this, less than 10% of Sox9 chondrocytes were tdTomato^+^ at 10 days (Fig. 7c). However, when we examined ectopic masses in skeletal muscle 7-days after BMP2-injection, on average ~48% of Sox9^+^ chondrocytes in Safranin-O stained cartilage was also tdTomato^+^ (Fig. 7d). To further confirm that Clec3b^+^ cells can differentiate into chondrocytes, we digested the skeletal muscles of *Clec3b*-lineage reporter mice and purified primary tdTomato+ cells with flow cytometry. Following 21-days of chondrogenic pellet culture (in activation media containing Tgfβ3 and BMP6), we found that Clec3b^+^ cells gave rise to chondrocytes ex vivo. Thus, Clec3b^+^ cells make very limited contributions to cartilaginous activity during fracture repair; however, this is likely a context-specific limitation.

## Discussion

Here, we have shown that *Clec3b*-expression marks FAPs and superficial periosteal cells, which are osteogenically dormant under normal conditions but can be recruited to assist with the repair of bone fractures. Loss of function in Clec3b^+^ cells, through both targeted depletion and WNT-inhibition, reduces callus bone volume fraction during fracture repair (Fig. 6). While both FAPs^19^ and superficial periosteal cells^15^ have previously been implicated as osteoprogenitor pools activated by bone fracture, our drill hole-injury, mechanical loading and orthotopic transplantation experiments indicate that Clec3b^+^ FAPs are the likely source of bone formation rather than Clec3b^+^ periosteal cells (Fig. 3). We have independently verified the osteogenic potential of Clec3b^+^ FAPs through intramuscular BMP2-injection experiments (Figs. 5 and 6), which also show that depletion and WNT-inhibition in Clec3b^+^ cells significantly limits ectopic calcification of muscle.

Notably, the response of Clec3b^+^ cells to fracture is distinct from previously defined skeletal stem/progenitor populations in the periosteum, such as those marked by the expression of *Ctsk*,^3^ *Gli1*,^12^ *Pdgfra*^23^, or *Acta2*/aSMA,^13^ in that Clec3b-lineage cells exhibit minimal chondrogenic activity. Conversely, the aforementioned periosteal populations form >50% of chondrocytes during the initial 10 days post-injury in the mouse model of stabilized long bone fracture. *Clec3b*-expression is also absent in myogenic-lineage cells, unlike Hoxa11^+^ or Gli1^+^ cells, both of which contribute to myofiber formation in skeletal muscle. Our data confirm and expand the documented plasticity of stromal cells in muscle and peripheral periosteum, as Clec3b^+^ cells convert to osteoblasts and some CAR cells, in addition to adipocytes and fibroblasts upon perturbation, but not myogenic cells. Thus, Clec3b^+^ cells both complement (in terms of chondrogenesis) and partially overlap (with respect to fibroadipogenic progenitors) with previously described progenitor populations.

We found that Clec3b^+^ cells give rise to chondrocytes in the ankle joint following Achilles tendon injury (Fig. 5g); however, at least some of these cells are likely of connective tissue origin and might respond differently to injury. To determine whether Clec3b^+^ FAPs fundamentally lack the capability to differentiate into chondrocytes, we have examined their response to BMP2-stimulation during early stages of ectopic mineralization, a process mediated by endochondral ossification. We found that Clec3b^+^ FAPs give rise to a much larger fraction of Sox9^+^ chondrocytes in this model (Fig. 7d), which contrasts with their behavior post-fracture. Pellet cultures of Clec3b^+^ FAPs also showed that they can form cartilage ex vivo (Fig. 7e). Thus, the unique absence of chondrogenic activity by Clec3b^+^ cells in fracture repair is context-specific. This could be due to a lack of necessary molecular cues in vivo, which would drive cartilage formation (such as combined Tgfβ- and BMP-stimulation, an essential component of ex vivo chondrogenic differentiation assays^46^), or rapid response of periosteal cells before Clec3b^+^ cells could migrate to the callus and participate in chondrogenesis.

Importantly, 2 previous studies that characterized FAPs within the context of fracture repair suggested that FAPs exhibit a chondrogenic response to bone injury. Julien et al. reported that fluorescently labeled *Prrx1*-lineage cells, when grafted into the limbs of host mice, undergo chondrogenic differentiation following non-stabilized fracture injury.^19^ More recently, He et al. reported that Prg4^+^ cells (which represent a small subset of FAPs) are also mobilized in a stabilized fracture model and give rise to Col2a1^+^ chondrocytes in the fracture callus.^47^ However, no quantification was reported in either study (regarding neither recombination efficiency nor the overlap between FAP-lineage cells and chondrocytes), which makes it difficult to interpret the physiologic significance of FAP-derived chondrocytes described in these studies. Further research into this intriguing difference between Clec3b^+^ cells and other skeletal stem and progenitor cells could reveal the mechanisms that drive or inhibit chondrogenic differentiation of FAPs (and presumably other populations) during bone healing.

While we find that Clec3b^+^ FAPs make a significant contribution to fracture repair, disabling them does not cause a severe phenotype as seen in clinical nonunion cases. This could be due to the technical limitations in our approach such as recombination inefficiency, or that the absence of FAPs could eventually be compensated by other osteoprogenitor populations, such as periosteal stem cells. The recent work by He et al. also shows that depletion of the Prg4^+^ subset of FAPs similarly reduces bone volume fraction in the fracture callus (following a ~63% depletion of targeted cells, similar to our findings in Fig. 6).^47^ Collectively, these findings indicate the importance of muscle response to bone injury and implicate FAPs as a potential target for therapies aimed to enhance bone healing.

Heterotopic ossification is an abnormal bone formation event that involves concurrent osteogenic and adipogenic differentiation of progenitor cells, which might occur in response to trauma or genetic factors. While the physiologic mechanisms of disease are complex (involving the activation of neural and inflammatory pathways^48–51^) and may not be fully captured by BMP2-mediated stimulation, our data recapitulate the adipogenic and osteogenic cellular features of heterotopic ossification. Interestingly, diphtheria toxin-mediated ablation of Clec3b^+^ cells prior to BMP2 stimulation reduces heterotopic ossification, whereas continuous depletion after BMP2-treatment does not. Our results suggest that this discrepancy could be due to the osteogenic mobilization of tdTomato- cells in the latter case (either due to inefficient recombination or lack of *Clec3b*-expression), which contribute to a higher fraction of osteocytes following targeted cell ablation (Fig. S7e). Notably, we also found that the bone volume of the fracture calluses in mice serving as control for the cell ablation experiment are almost twice as high as the control samples in β-catenin-deletion experiment, likely due to repetitive tamoxifen treatment in the former case (Fig. 6). These differences highlight the technical limitations of targeted cell ablation approach in assessing functional relevance of Clec3b^+^ cells in bone formation.

The full extent of the mechanistic differences between Clec3b^+^ cells and other populations in different contexts (e.g., fracture healing vs. heterotopic ossification) remains to be determined. However, our findings show that Clec3b^+^ cells (and likely their FAP subset) are important contributors to fracture healing in a WNT-dependent manner. Understanding the pathways that regulate Clec3b^+^ cells and their fate decisions may suggest new strategies to treat patients suffering from musculoskeletal trauma.

## Materials and methods

### Generation of the Clec3b^CreERT2^ allele

All experiments were approved by the Institutional Animal Care and Use Committee of Weill Cornell Medicine (protocol#: 2018-0019). Clec3b^creERT2^ mice were generated at the Mouse Genetics Core Facility at Memorial Sloan Kettering Cancer Center by modifying the tetranectin protein, through insertion of a T2A-CreERT2 construct at the 3′ end of Clec3b endogenous coding sequence in embryos. A total of 128 pups on a C57 background were screened with PCR, and 2 mice (1 male and 1 female) were identified as potential founders with the correct insertion of the construct (Fig. S2). Both mice were crossed to male or female conditional reporter mice, and their F1 progeny were found to yield identical recombination profiles when treated with tamoxifen.

### Additional mice

Gt(ROSA)26Sor^tm14(CAG–tdTomato)Hze^/J (RRID:IMSR_JAX:007914), B6.129P2-Gt(ROSA)26Sor^tm1(DTA)Lky^/J (RRID:IMSR_JAX:009669), B6.129-Ctnnb1^tm2Kem^/KnwJ (RRID:IMSR_JAX:004152), B6.129S4-*Pdgfra*^*tm11(EGFP)Sor*^/J (RRID:IMSR_JAX:007669) and Gt(ROSA) 26Sor^tm1(Smo/EYFP)Amc^/J (RRID:IMSR_JAX:005130) mice were purchased from Jackson Laboratory (Bar Harbor, ME, USA). C57BL/6-Tg(BGLAP-Topaz)1Rowe/J mice (RRID:IMSR_JAX:017469^52^) were provided by Dr. Noriaki Ono. Cxcl12-eGFP mice (RRID:MGI:4838648^53^) were provided by Dr. Takashi Nagasawa. All mice were maintained in standard housing conditions, PCR-genotyped at 14 days, and weaned at 21 days. To induce Cre-mediated recombination, we administered tamoxifen (cat# T5648, Sigma, St. Louis, MO; 75 mg/kg dissolved in corn oil) three times to each mouse intraperitoneally, at P21, P23, and P25, unless indicated otherwise. Mice were euthanized with slow exposure to CO_2_ at the indicated time points. Unless specifically noted, both male and female mice were used in all experiments.

### Fracture surgeries

Six-week-old mice were anesthetized with continuous isoflurane inhalation. The fur around the surgical site was shaved and the skin was sterilized with betadine and ethanol. Bupivacaine was injected subcutaneously for local anesthesia. The right femoral diaphysis was exposed with a 2 cm incision along the skin and muscle. Fractures were created by performing a mid-diaphysis osteotomy with a high-speed saw, followed by the insertion of a 25 G sterile stainless-steel pin into the intramedullary cavity. Pin insertion was not performed in unstabilized fracture surgeries. The skin was closed with surgical clamps. The mice received meloxicam (2 mg/kg, at the time of surgery and every 24 h for 3 days) and buprenorphine (0.5 mg/kg, at the time of surgery and every 12 h for 3 days) subcutaneously for pain relief. The correct positioning of the intramedullary pins was verified with X-ray imaging immediately after surgery, and prior to euthanasia.

### Drill hole surgeries

Mice were prepared for surgery as described above. The right femoral diaphysis was exposed, and the periosteum was either left intact or carefully scraped with a scalpel blade along a ~2 cm surface around the intended injury area. Monocortical drill holes were then created with a high-speed drill equipped with a 0.9 mm drill burr. The skin was closed with surgical clamps, and the specimens were collected 3 weeks later for analysis.

### Femoral transplant surgeries

Mice were prepared for surgery as described above. Immediately before the procedure, Clec3b^creERT2/+^; R26^Ai14.tdTomato/+^ mice were euthanized and the central ~2 mm portion of the femoral diaphysis was removed either with or without surrounding muscle, the bone marrow was flushed and placed in cold sterile PBS. A ~2 mm defect was created in same-sex littermate mice that lack the Clec3b^creERT2^ allele, and the gap was filled with the graft and a 25 g sterile stainless-steel pin that aligned the graft with the native femur. The site of injury was closed and the mice were treated and monitored as described in fracture surgeries.

### Heterotopic ossification models

Recombinant human BMP-2 (Cat#Z02913, GenScript, Piscataway, NJ) was dissolved in 20 µmol/L acetic acid to create a 0.1 µg/µL stock solution. For each injection, 0.25 µg of BMP-2 stock was mixed with 25 µL of Matrigel (Cat#CLS356231, Corning, Corning, NY). The resultant mixture was injected into the right caudal thigh muscle of 5-week-old mice. Muscle and bone specimens were collected for analysis 3 weeks later. For tenotomy, 5-week-old mice were anesthetized with isoflurane, and the right ankle joint was shaved and sterilized as described above. The Achilles tendon was exposed with a scalpel and forceps, and severed at mid-point with sharp scissors. Skin was closed with surgical clamps, and mice were monitored with Faxitron for signs of calcaneus remodeling and ectopic calcification on a bi-weekly basis. Mice were sacrificed for specimen collection at 8- or 10-week post-surgery.

### In vivo mechanical loading

Ten-week-old *Clec3b*^*creERT2/+*^*; R26*^*Ai14.tdTomato/+*^ mice were sedated with isoflurane. The left tibia was positioned inside a custom-designed fixture (as previously described^54,55^) and loaded with 7.0 N peak load at 4 Hz for 1 200 cycles (i.e., 5 min of loading total) 5 days per week for 2 weeks. Tibial specimens were collected at the end of 2-week period.

### Flow cytometry and single-cell RNA-seq

Femoral fracture calluses and intact diaphyses with surrounding muscle were collected from *n* = 3 littermate male mice 3 weeks post-fracture surgery. Following dissection with scissors and forceps, fracture and intact specimens were separately pooled inside petri dishes with sterile PBS on ice. The specimens were then moved to a tissue-culture hood, finely cut with a razor blade, and put in a 15 mL tube with 10 mL collagenase solution (4 000 units in αMEM with 1% anti-mycotic; type II, Worthington Biochemical Corp, Lakewood, NJ, USA). The tubes were transferred to a shaking incubator for 2 × 30 min at 37 °C. Cells were filtered, pelleted, and resuspended in cell culture media (αMEM with 10% FBS and 1% anti-mycotic). Cells were tested for viability with an automated fluorescent cell counter and then sorted on an Aria II flow cytometer (BD Biosciences, San Jose, CA, USA) to separate tdTomato-expressing cells (with cells from wild type mice used as negative control). The purified tdTomato^+^ cells were loaded on the 10× Chromium platform and processed for single-cell RNA-seq using the 3′ single-cell RNA sequencing kit V3. The resultant libraries were sequenced on the Illumina NovaSeq platform and processed with Cellranger and Seurat pipelines. All analyses were performed in R with custom scripts (available upon request). Gene set enrichment analysis (GSEA) was performed with previously published tools.^56,57^ For T2A-CreERT2 construct verification, data were processed with the de novo assembly tool Trinity on Galaxy (ver 2.15.1).

### Fluorescence-activated cell sorting (FACS)

Muscle and/or bone samples were carefully dissected immediately after euthanasia, and live cells obtained as described above. Cells were resuspended in FACS buffer (2% FBS + 1% anti-mycotic in PBS) and incubated with conjugated primary antibodies CD45-PE-Cy7 (BioLegend Cat#103113, RRID:AB_312979), CD31-PE-Cy7 (BioLegend Cat#102524, RRID:AB_2572181), TER119-PE-Cy7 (BioLegend Cat#116221, RRID:AB_2137789), Sca-1 (Thermo Fisher Scientific Cat# 17-5981-81, RRID:AB_469486) and DAPI (Sigma Cat# 10236276001) for 1 hour on ice. Cells were analyzed on the Symphony platform (BD Biosciences, San Jose, CA, USA). Compensation was calculated with antibody-treated beads and gates were set with respect to unstained cells and fluorescence-minus-one negative controls.

### Ex vivo chondrogenic differentiation

Primary tdTomato^+^ cells were purified from the skeletal muscles of 3–4-week-old Clec3b-lineage reporter mice as described above (*n* = 2 mice per experiment), collectively yielding 20 000–40 000 cells. Cells were then plated in 6-well plates and allowed to reach confluence and passaged at least twice, while changing media every 2–3 days. Cells were trypsin-digested, counted, and split into groups of 250 000 cells/tube. Cells were pelleted with centrifugation inside 15 mL tubes and chondrogenic differentiation was stimulated using the Chondrogenic SingleQuots Kit (Cat#PT-3003, Lonza), supplemented with TGFβ-3 (10 ng/mL, cat#:PT-4124, Lonza) and BMP-6 (500 ng/mL, cat#6325-BM-020/CF, R&D Systems). Media was changed every 2–3 days, and pellets were embedded in OCT 21-days later for histologic analysis.

### Histology

Following euthanasia, the bone and/or muscle specimens were carefully dissected. The intramedullary pins were gently removed from the fracture calluses without disturbing their integrity. The specimens were placed in ice-cold 4% paraformaldehyde, with gentle overnight shaking at 4 °C. The specimens were then washed with cold PBS 3 times for >1 h and transferred to 0.5 mol/L EDTA for decalcification for 6–10 days. The specimens were washed and then saturated with cryoprotective 30% sucrose solution. The specimens were embedded in OCT and sections of 20 µm thickness were cut with a cryostat (CM3050 S, Leica, Nussloch, Germany). Every third slide was stained with DAPI and closed with Fluoroshield (Sigma #1123585236), with the remaining slides used for hematoxylin & eosin or safranin-O staining. All slides were imaged with a slide scanner (Axioscan 7, Zeiss, Oberkochen, Germany), and select slides were scanned with a confocal microscope (LSM 880, Zeiss) for high-resolution imaging. The images were adjusted for brightness and contrast with ImageJ.

### Immunohistochemistry

Histology slides maintained at −80 °C were allowed to thaw at room temperature for 20 min. Sections were rehydrated with PBS for 15 min, treated with 0.3% Triton solution (150 µL/slide, Sigma) for 15 min, and washed with PBS for 5 min. Sections were blocked with 5% donkey serum and incubated at room temperature for 30 min. Primary antibodies for Pdgfra (Abcam Cat#203491, RRID:AB_2892065), Sca1 (Abcam Cat#51317, RRID:AB_1640946), CD31 (BD Biosciences Cat#553370, RRID:AB_394816), perilipin (Cell Signaling Technology Cat#9349, RRID:AB_10829911), Sox9 (R&D Systems, Cat#AF3075, RRID:AB_2194160) and/or laminin (Abcam, Cat#11575, RRID:AB_298179) (1:100 dilution) were then applied and sections were allowed to incubate at 4 °C overnight enclosed with a damp paper towel. The next day, three PBS washes were performed at room temperature and sections were incubated with 150 µL of A488- or AF647-conjugated secondary antibody (Jackson Immunoresearch Labs Cat#705-545-147, RRID:AB_2336933; Cat#711-605-152, RRID: AB_2492288 1:400 dilution) for 3 h. Three PBS washes were performed at room temperature, sections were stained with DAPI, and closed with a coverslip. Secondary-only negative controls were used to verify the specificity of the primary antibodies.

### In situ hybridization

Hindlimb muscles were collected from C57/BL6 mice and immediately frozen in liquid nitrogen. Muscle specimens were embedded in OCT, sectioned at 20 μm-thickness as described above and maintained at −80 °C. Slides were processed using the RNAscope HiPlex12 Reagent Kit (v2, Cat#324419, Advanced Cell Diagnostics). Briefly, fresh frozen muscle sections were fixed in pre-chilled 4% PFA for 1 h at 4 °C. Slides were dehydrated sequentially in 50%, 70% and 100% ethanol, and stored inside 100% ethanol overnight at −20 °C. Slides were then permeabilized through incubation with the Protease IV reagent for 30 min, and then hybridized with probes for Clec3b (cat#539561-T2) and Pdgfra (cat#480661-T3) or positive/negative controls at 40 °C for 2 h, followed by RNAscope HiPlex assay. Slides were additionally treated with the FFPE reagent in order to reduce autofluorescence. All slides were stained with DAPI, closed with coverslips and stored in the dark at 4 °C. Imaging was performed with a fluorescent microscope (Axio Observer 7, Zeiss, Oberkochen, Germany) and a confocal microscope (Stellaris 8, Leica Microsystems, Deerfield, IL).

### Micro-CT analysis

Bone and/or muscle specimens were dissected and scanned immediately after euthanasia. Samples were scanned at 7.4 μm resolution (55 kVp, 145 mA, 8 W) using the Scanco CT 45 platform (SCANCO, Bruttiselen, Switzerland). Identical thresholds were used in the segmentation of bone from different groups when quantifying mineralization. Fracture calluses were analyzed by selecting region of interest as 1 mm proximal and distal (2 mm in total) from the center. Total volume (TV), bone volume (BV) and bone volume fraction (BV/TV) parameters were calculated as previously described.^58^

### qRT-PCR

Total RNA was extracted from primary cells (directly sorted into Trizol) or snap-frozen muscle tissue through phenol-chloroform separation, followed by purification with the Purelink RNA Mini Kit (cat#12183018A, Thermo Fisher) and DNase treatment (cat#79254, Qiagen). RNA quality was assessed with Nanodrop, and reverse transcription was performed with the Superscript III kit (cat#: 18080093, Thermo Fisher). The resultant cDNA was amplified with primers for *Clec3b* (Fwd: CTGAAGGAGAAGCAGGCCT, Rev: GCCTCGTTCTCTAGCTCTGA) or *E11* as the housekeeping gene (Fwd: GATCAAGATGTGGACCGTGC, Rev: AGGTGCCTTGCCAGTAGATT), using the PowerUp SYBR Green Master Mix (cat#A25741, Thermo Fisher). Relative expression of *Clec3b* was quantified with respect to *E11*, using the C_t_ values measured for each sample.

## Supplementary information

Supplementary Figures

## Data Availability

The single-cell RNA-seq data were uploaded to the Gene Expression Omnibus (GEO) database under the accession number GSE249546.
