# Supplementary material for: Clec3b+ extraskeletal cells regulate fracture healing and heterotopic ossification
Source: Bone Res. 2026 Jul 6;14:70. doi: 10.1038/s41413-026-00532-6 (PMC13338248; doi:10.1038/s41413-026-00532-6)
Supplement: Supplementary file 1 — Supplementary Figures [file 41413_2026_532_MOESM1_ESM.docx]

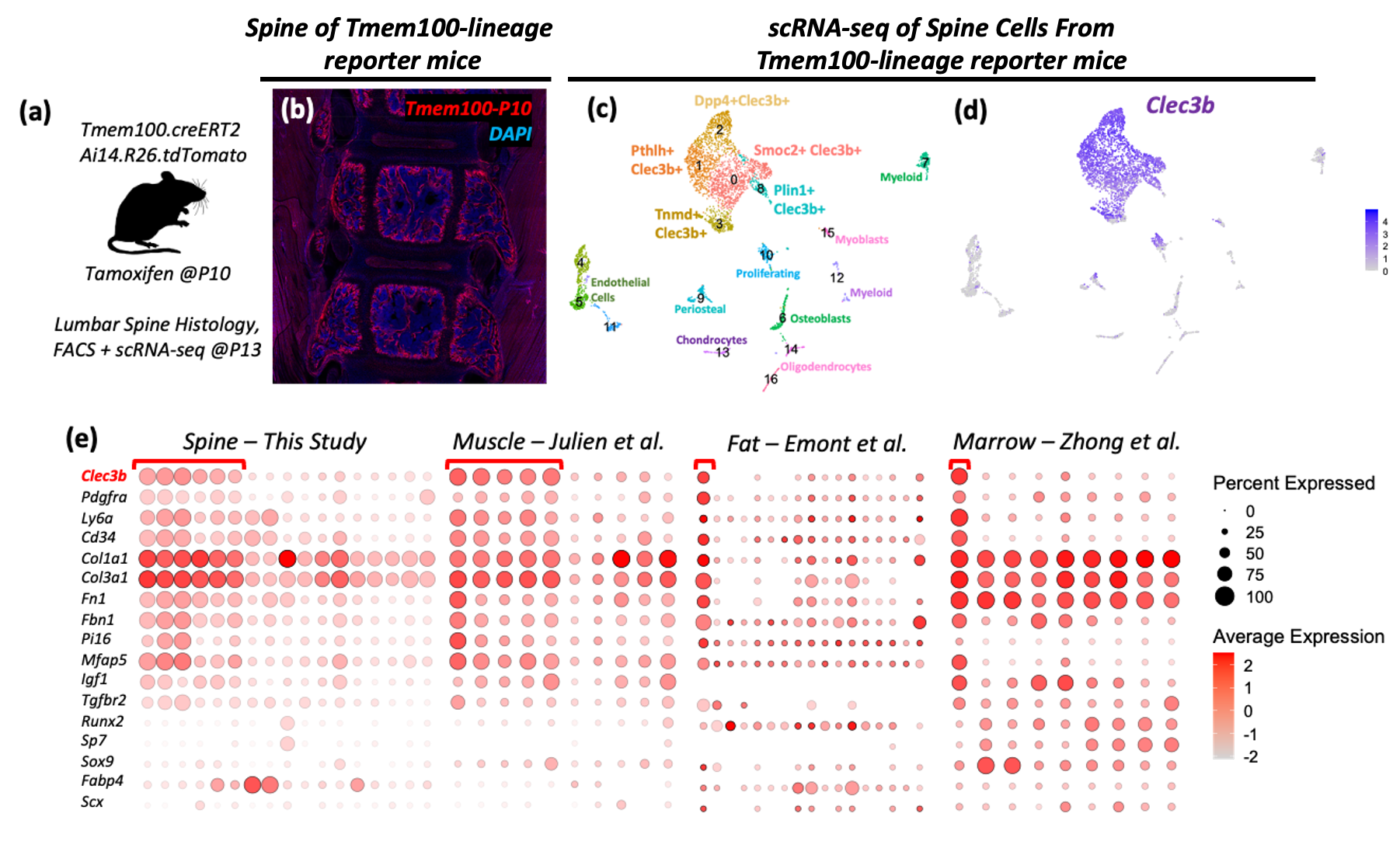


**Supplementary Figure 1: Clec3b expression is specific to Pdgfra+ and Ly6a+ cells in multiple tissue types.**

**(a)** Lumbar spine specimens from Tmem100-lineage reporter mice were obtained for single cell RNA-seq analysis 3 days post-tamoxifen treatment.

**(b)** Histology confirms that Tmem100 is a broad marker for mesenchymal lineage cells, as most bone-lining surfaces within the vertebrae, surrounding muscle, connective tissue and some nucleus pulposus cells are tdTomato+.

**(c)** Single cell RNA-seq of tdTomato+ cells sorted from digested spine tissue indicates a heterogeneous pool of cells that include Clec3b+ cells (with subsets distinguished by Smoc2, Pthlh, Dpp4, Plin1 and Tnmd expression) osteoblasts and endothelial cells.

**(d)** Clec3b-expressing cells collectively constitute the largest group of tdTomato+ cells.

**(e)** A dot-plot comparison of transcripts specifically expressed by Clec3b+ cells (indicated with a red bracket above each sub-panel) show a similar gene expression reportoire across tissues. Columns represent individual cell clusters, dot size represents the fraction of cells expressing each gene and color intensity represents average expression level.


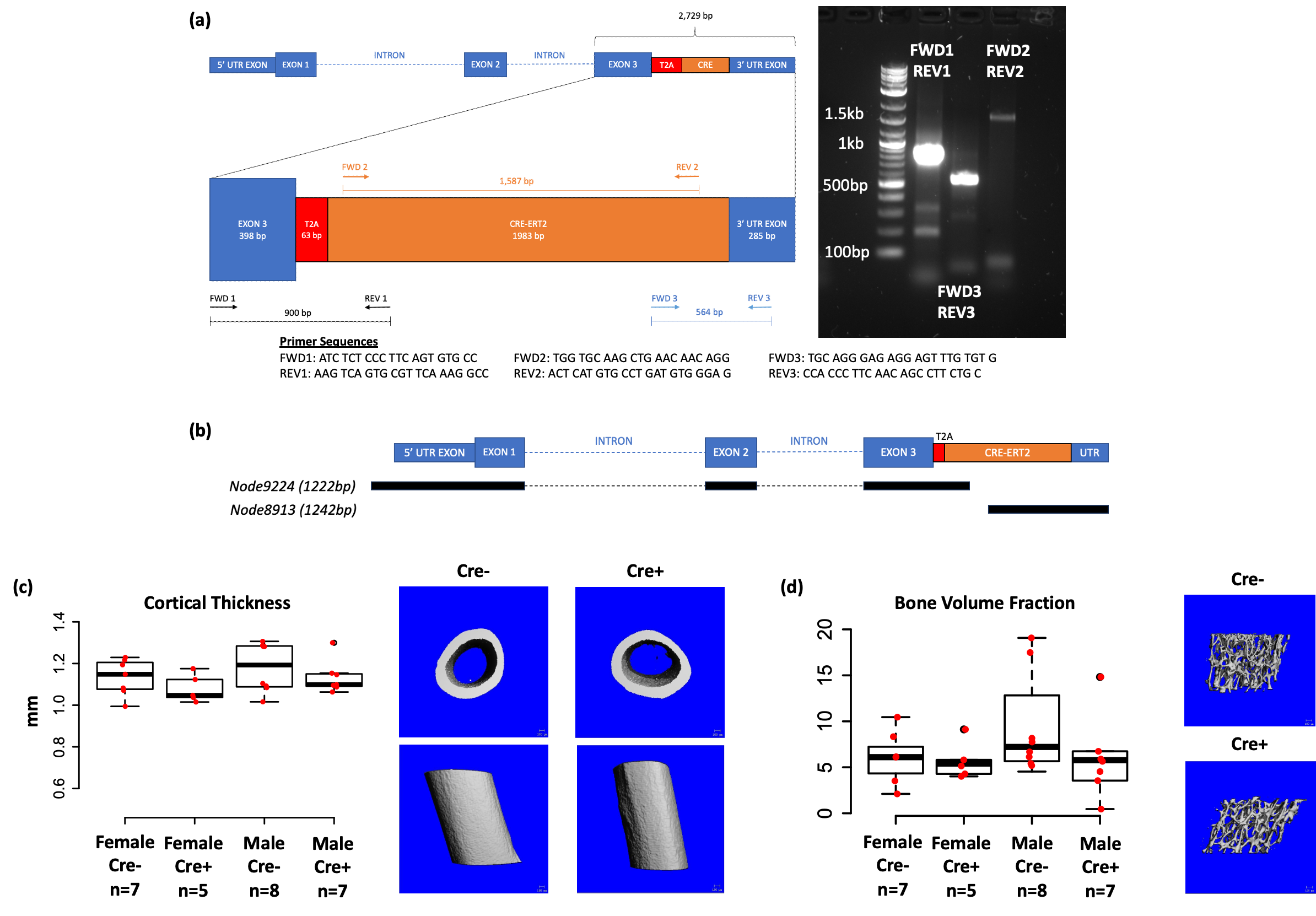


**Supplementary Figure 2: PCR and Sanger sequencing results indicate correct insertion of the T2A construct at the endogenous Clec3b locus, and no cortical bone differences are found between Cre+ and Cre- littermate mice.**

**(a)** The T2A - CreERT2 construct is inserted into the endogenous Clec3b-locus, between the coding and untranslated regions at the 3’ end. The construct was amplified from genomic DNA with the depicted primer pairs and correct sequence verified with Sanger sequencing.

**(b)** De novo transcriptome assembly with Trinity (using single cell RNA-seq data in Figure 4 as input) verified the expression of the construct, through the identification of 2 transcripts spanning the T2A and CRE-ERT-UTR loci.

**(c)** Diaphyseal cortical thickness of the femur was measured with µCT-imaging in male and female littermate mice, with or without the CreERT allele, at 9 weeks of age (*p* = 0.56, one way ANOVA). Representative 3D reconstructed images from Cre+ and Cre- mouse femurs are depicted.

(**d**) Trabecular bone in the distal femoral area was analyzed and no differences were found in terms of bone volume fraction (BV/TV), except for a trend (p=0.07) between male and female WT mice (one way ANOVA with post hoc Tukey).


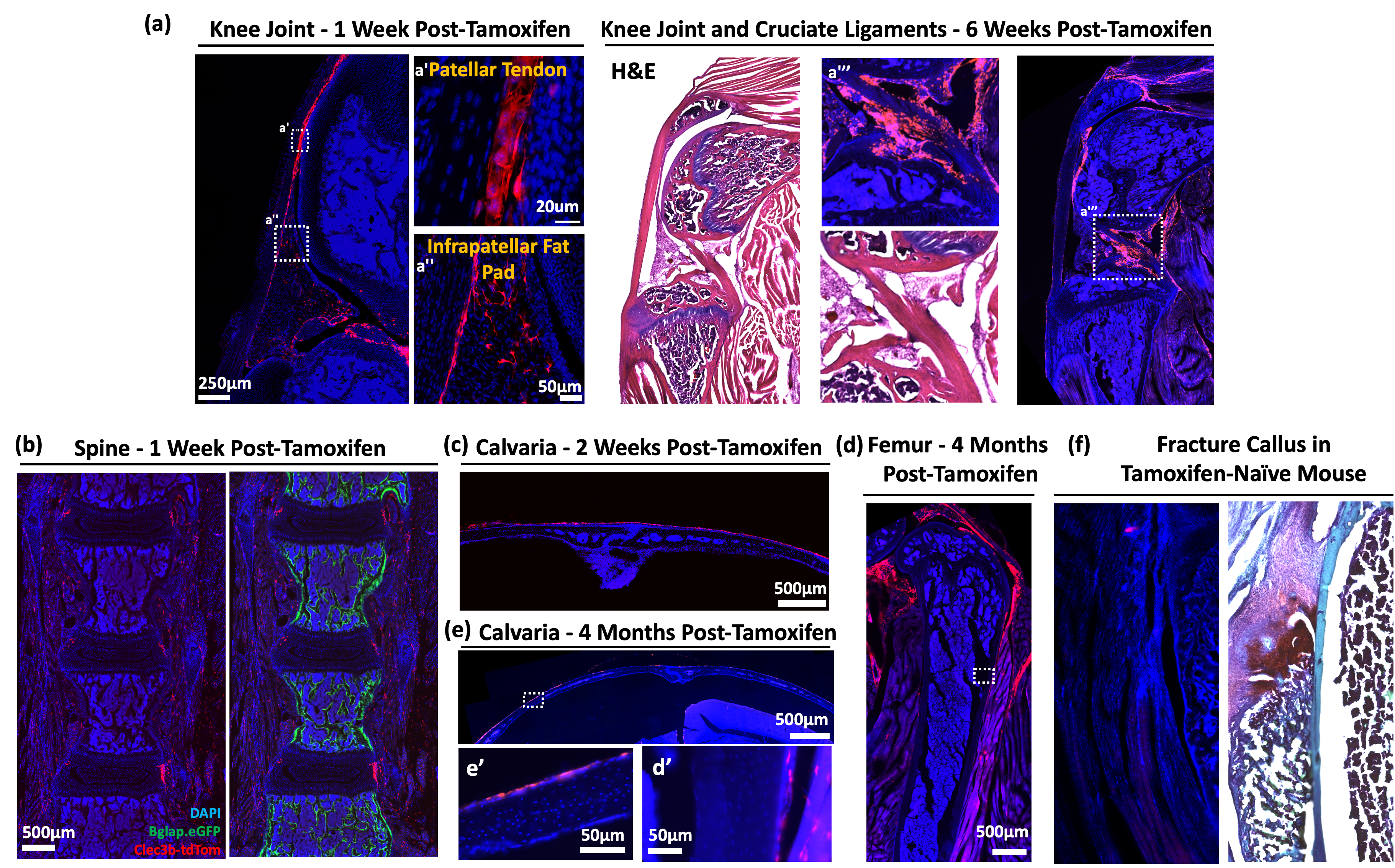


**Supplementary Figure 3: Tamoxifen-induced Cre-recombination results in the labeling connective tissue and superficial periosteum cells that remain osteogenically inactive.**

**(a)** Clec3b-tdTomato+ cells are found in the epitenon tissue in the patellar tendon and cruciate ligaments, as well as the infrapatellar fat pad stroma.

**(b)** Similar to the limbs, Clec3b-tdTomato+ cells are found in the muscles surrounding spine, and do not overlap with Bglap.eGFP+ osteoblasts.

**(c)** Clec3b-tdTomato+ cells are found in the superficial periosteum in the calvaria.

**(d and e)** Clec3b-tdTomato+ cells remain osteogenically inactive during the 4 months post-tamoxifen injection, as indicated by the lack of tdTomato+ osteocytes both in the femur and parietal bones.

**(f)** Fluorescent and safranin O-stained sections depict absence of tdTomato+ cells three weeks post fracture, when mice are kept tamoxifen-naïve.


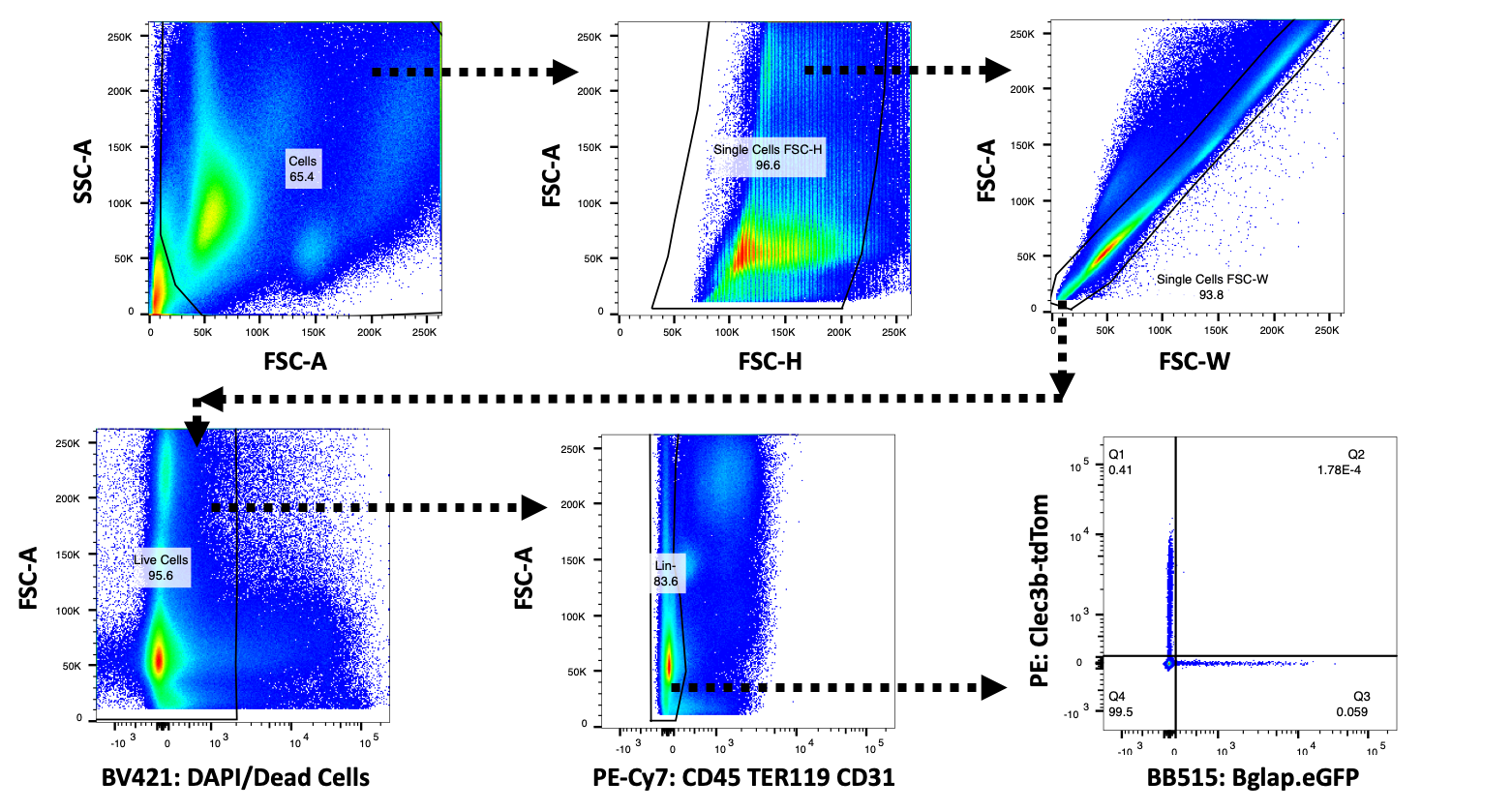


**Supplementary Figure 4: Representative plots depicting FACS strategy.**


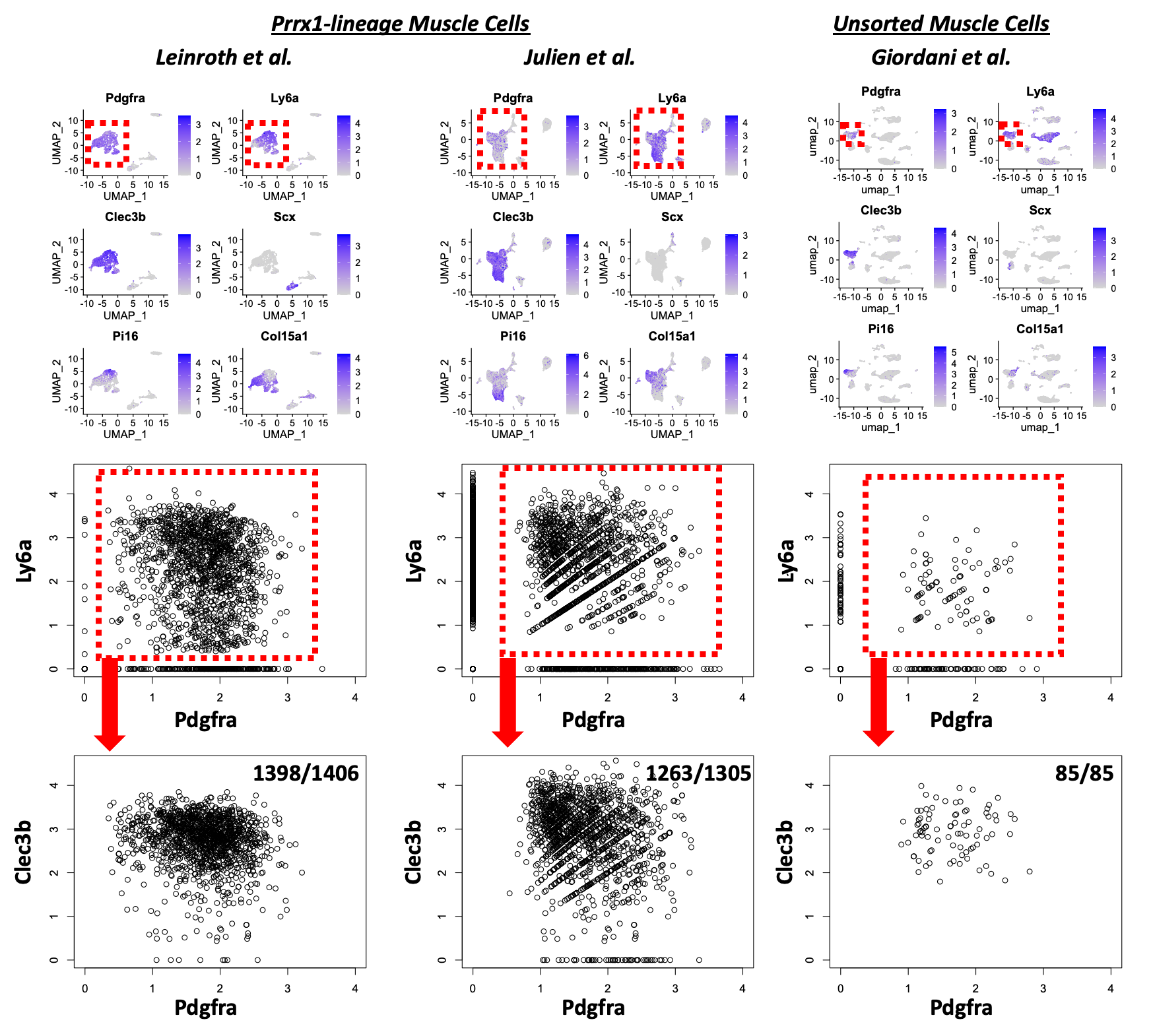


**Supplementary Figure 5: Analysis of previously published muscle single cell RNA-seq data.** Data were obtained from Prrx1-lineage muscle cells (from Leinroth et al., Cell Rep, 2022 and Julien et al., Nat Commun, 2021) and unsorted muscle cells (from Giordani et al., Mol Cell, 2019). In all 3 datasets, FAP clusters were identified based on co-expression of Pdgfra and Ly6a/Sca1. Cells with non-zero expression of both Pdgfra and Ly6a were also interrogated for Clec3b-expression, wherein almost all Pdgfra+ Ly6a+ cells were found to be Clec3b+.


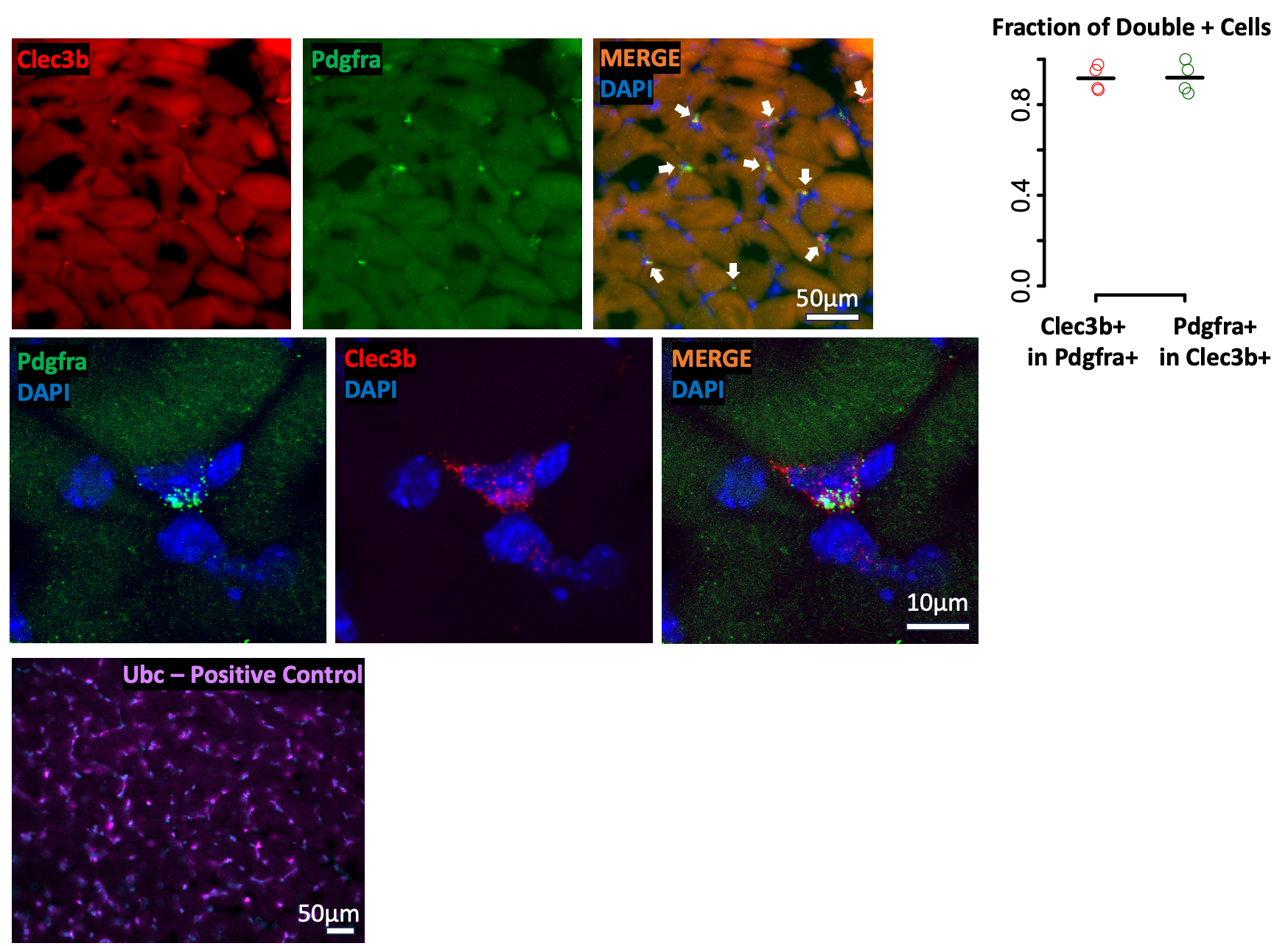


**Supplementary Figure 6: RNAscope analysis demonstrates overlap of Pdgfra+ and Clec3b+ cells in mouse skeletal muscle.** Hindlimb muscles of 3-4 week old male and female mice (n=4) were hybridized with probes recognizing *Clec3b* and *Pdgfra* mRNA. >90% of all Pdgfra+ cells were also Clec3b+ and vice versa. High magnification images depict individual Pdgfra and Clec3b mRNA molecules found in an interstitial muscle cell. Bottom panel depicts Ubc expression, which was used as a positive control.


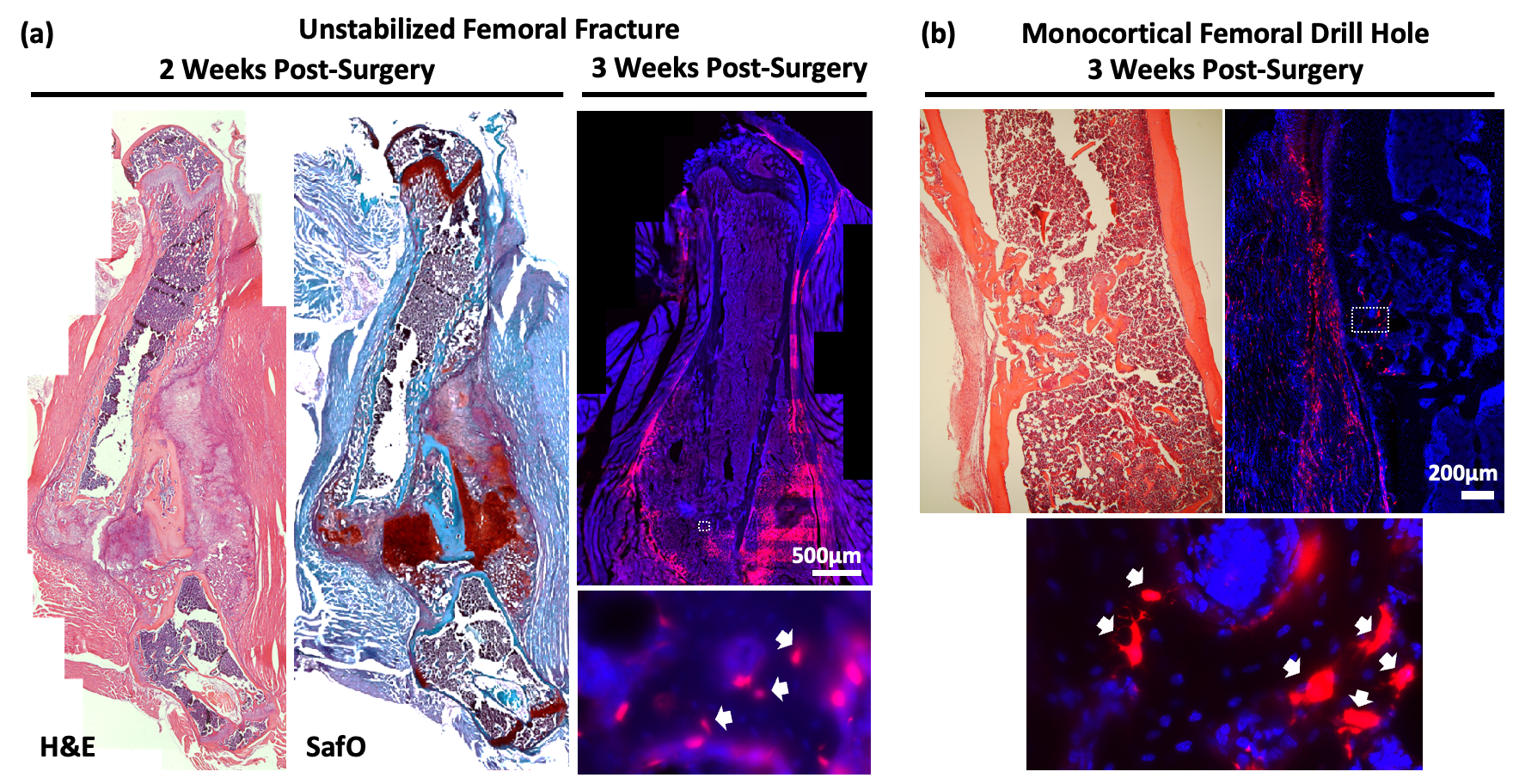


**Supplementary Figure 7: Monocortical drill hole and full osteotomy of the femoral diaphysis (i.e. unstabilized femoral fracture) activate osteogenic differentiation of Clec3b+ cells.**

**(a)** Representative histology images depict the formation of a large fibrocartilage callus following surgical induction of a femoral fracture that is not stabilized with an intramedullary pin. At 3 weeks, Clec3b-lineage tdTomato+ cells give rise to bone-lining cells and embedded osteocytes (arrows).

**(b)** Clec3b-lineage tdTomato+ cells appear to contribute to the healing of monocortical drill hole injuries to a lesser extent; however some tdTomato+ cells still become osteocytes (arrows).


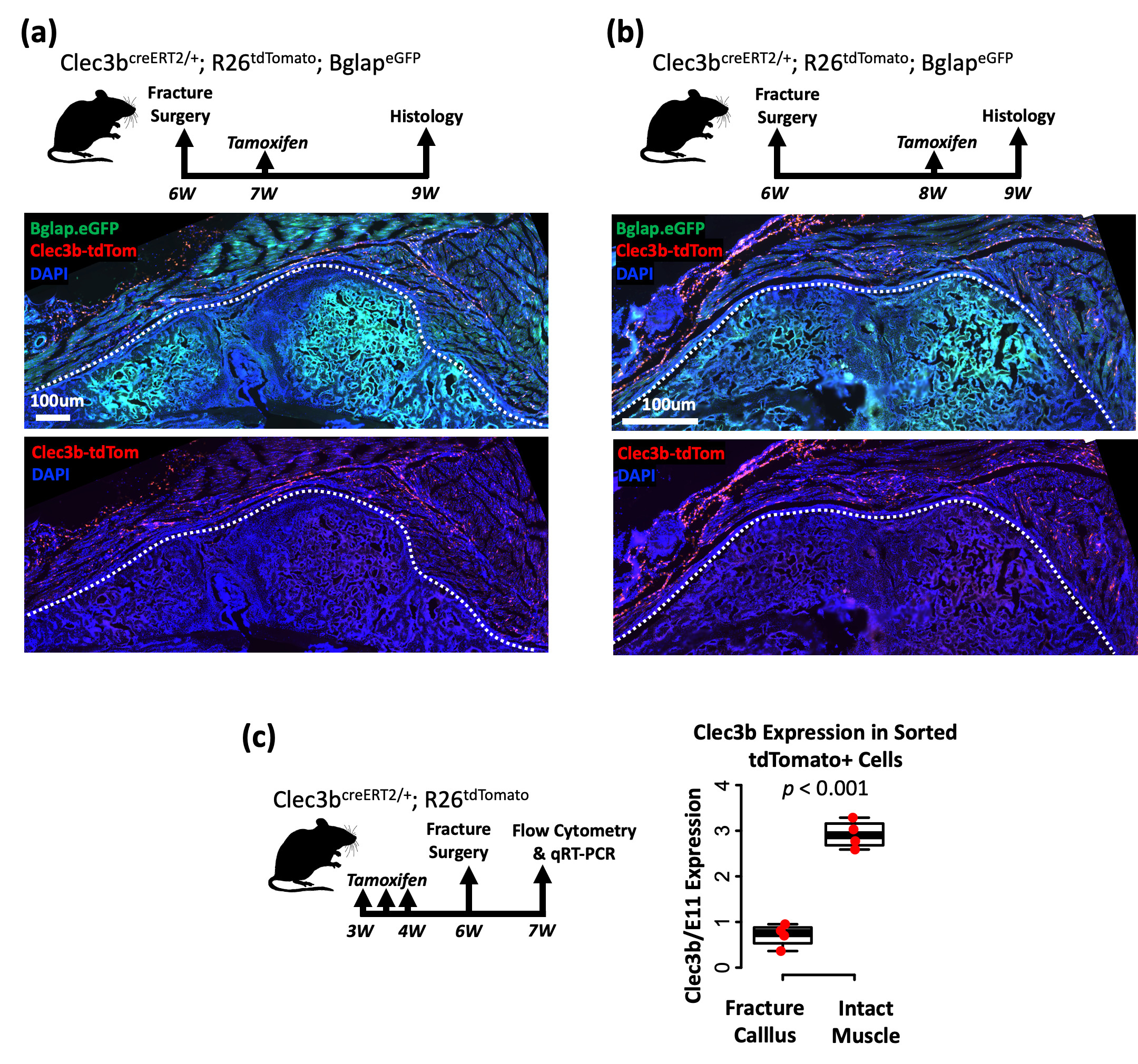


**Supplementary Figure 8: Tamoxifen treatment after fracture surgery does not label callus cells. (a)** Clec3b-lineage reporter mice were injected with tamoxifen 1 week after fracture surgery. No tdTomato+ cells were found in the fracture callus at 3 weeks. **(b)** Similar to (a), mice were treated 2 weeks after fracture surgery; however, no tdTomato+ cells were found in the callus at 3 weeks. **(c)** Two weeks after tamoxifen treatment, Clec3b-lineage reporter mice were subjected to fracture surgery. One week later, tdTomato+ cells were purified from the fracture callus and intact muscle in the contralateral limb for qRT-PCR, which revealed a significant depletion of Clec3b-expression in callus tdTomato+ cells.


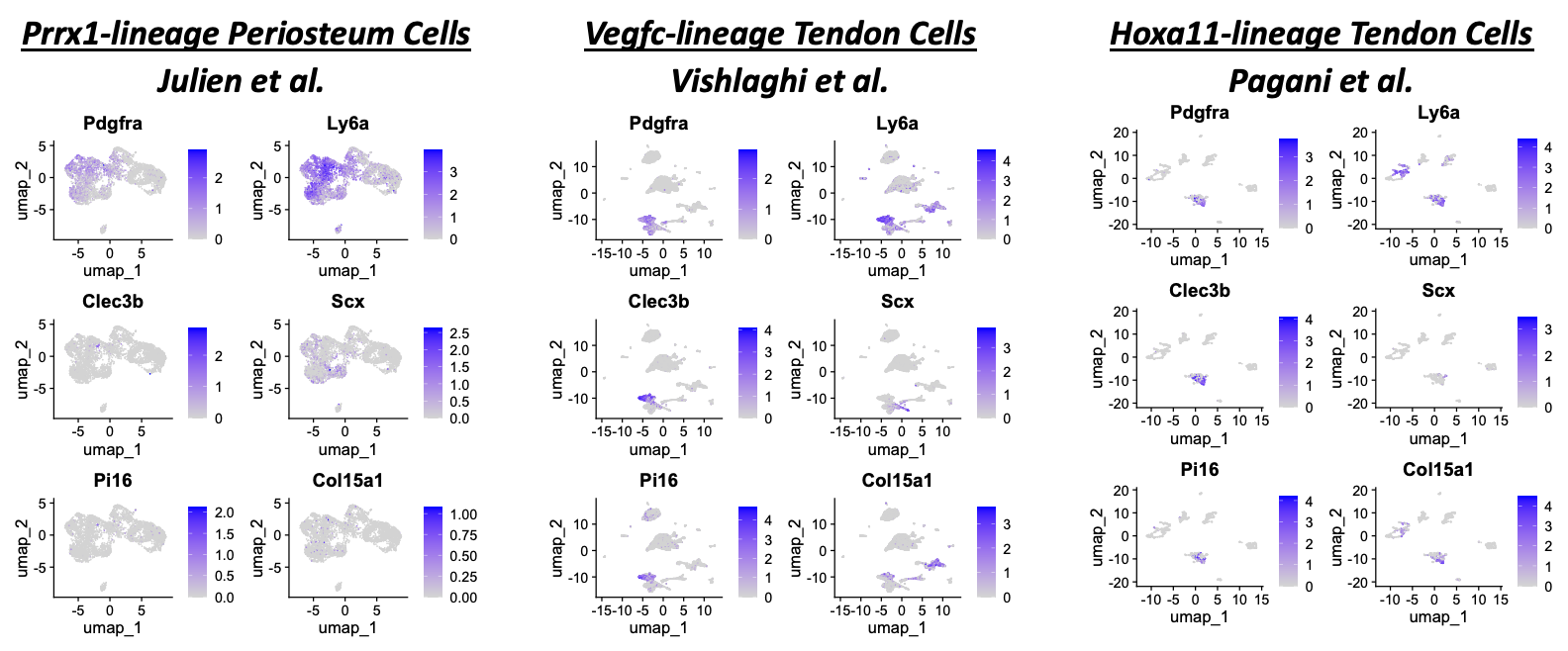


**Supplementary Figure 9: Single cell RNA-seq data obtained from periosteum and connective tissue by other investigators were analyzed for marker transcripts associated with Clec3b.** In all datasets, Clec3b is consistently a marker for Pdgfra+ Ly6a/Sca1+ cells, with Col15a1+ or Pi16+ subsets. Notably, few Clec3b+ cells are identified in the periosteum, and Clec3b+ cells do not overlap with Scx+ cells. Data were obtained from Prrx1-lineage periosteum cells (from Julien et al., J Bone Miner Res, 2022), Vegfc-lineage tendon cells (from Vishlaghi et al., Cell Rep, 2024) and Hoxa11-lineage tendon cells (from Pagani et al., Stem Cell Reports, 2021).

**
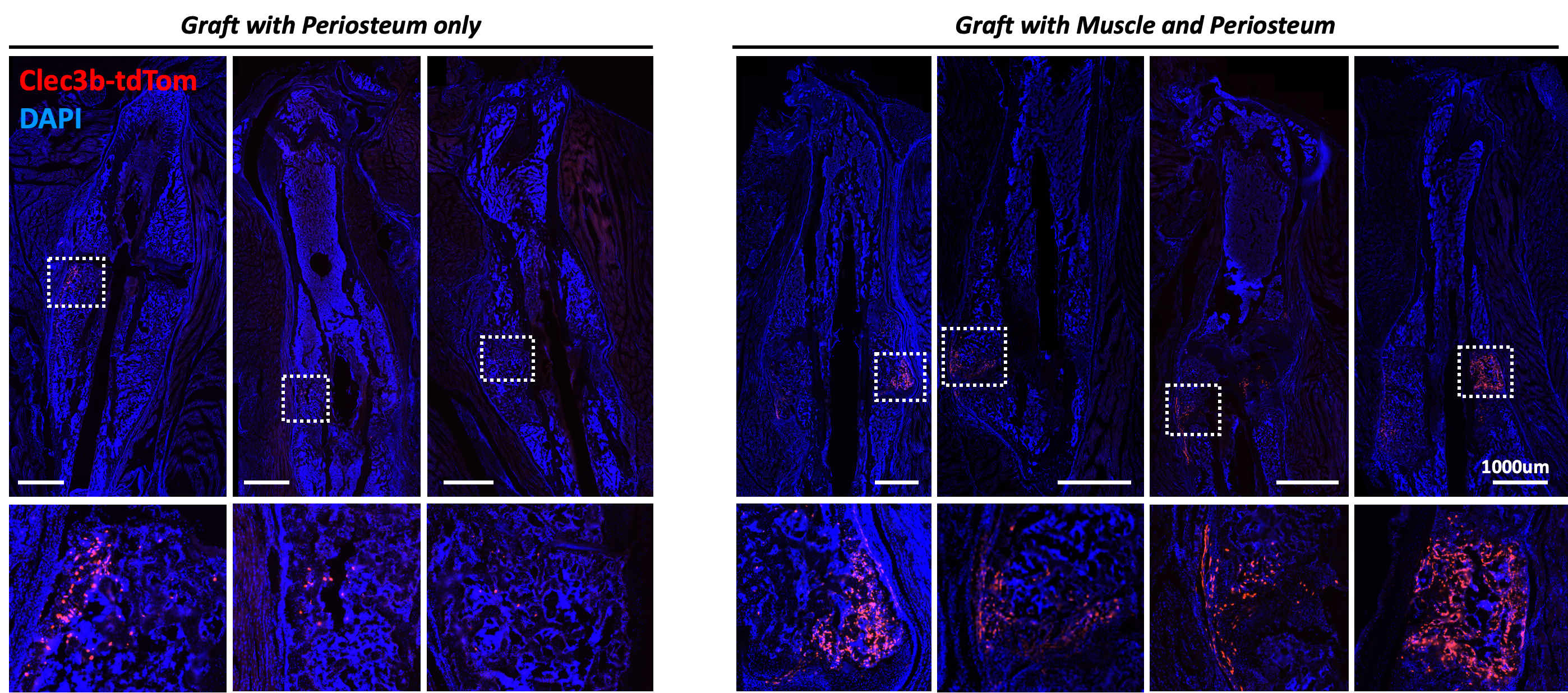
**

**Supplementary Figure 10: Images from additional recipient mice (3 weeks post-transplantation surgery) used in the quantification analysis depicted in Figure 3.**


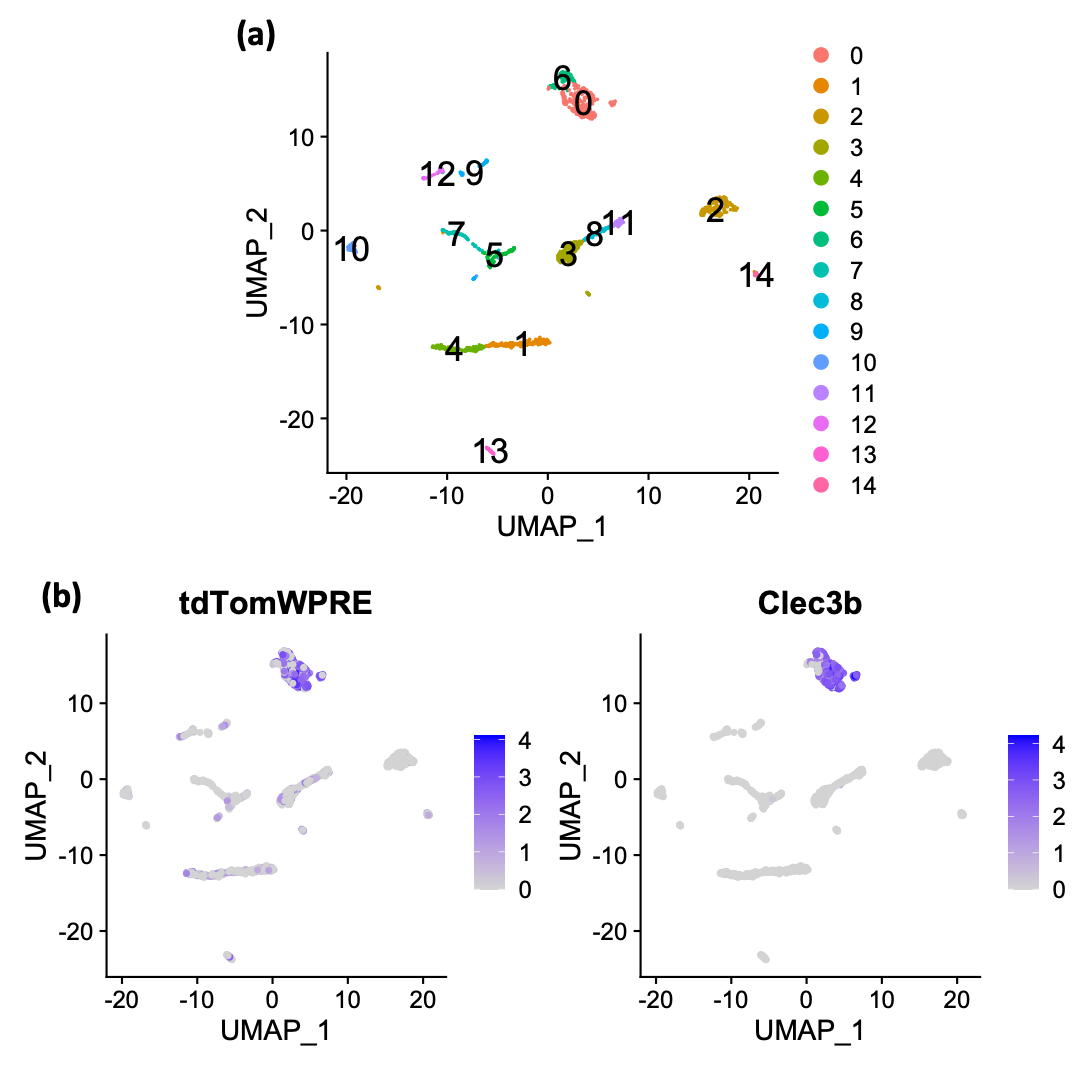


**Supplementary Figure 11: Single cell RNA-seq analysis shows lack of tdTomato expression in non-stromal populations.**

1. UMAP analysis of single cell RNA-seq data obtained from the intact muscle of Clec3b^CreERT2^; R26^tdTomato^ mice.
2. Dotplots show that tdTomato expression is limited to Clec3b+ cells.

**Supplementary Figure 12: Expression of skeletal stem cell marker transcripts (described by Chan et al. Cell, 2015), and the proliferation marker Mki67, in Clec3b+ cells.**


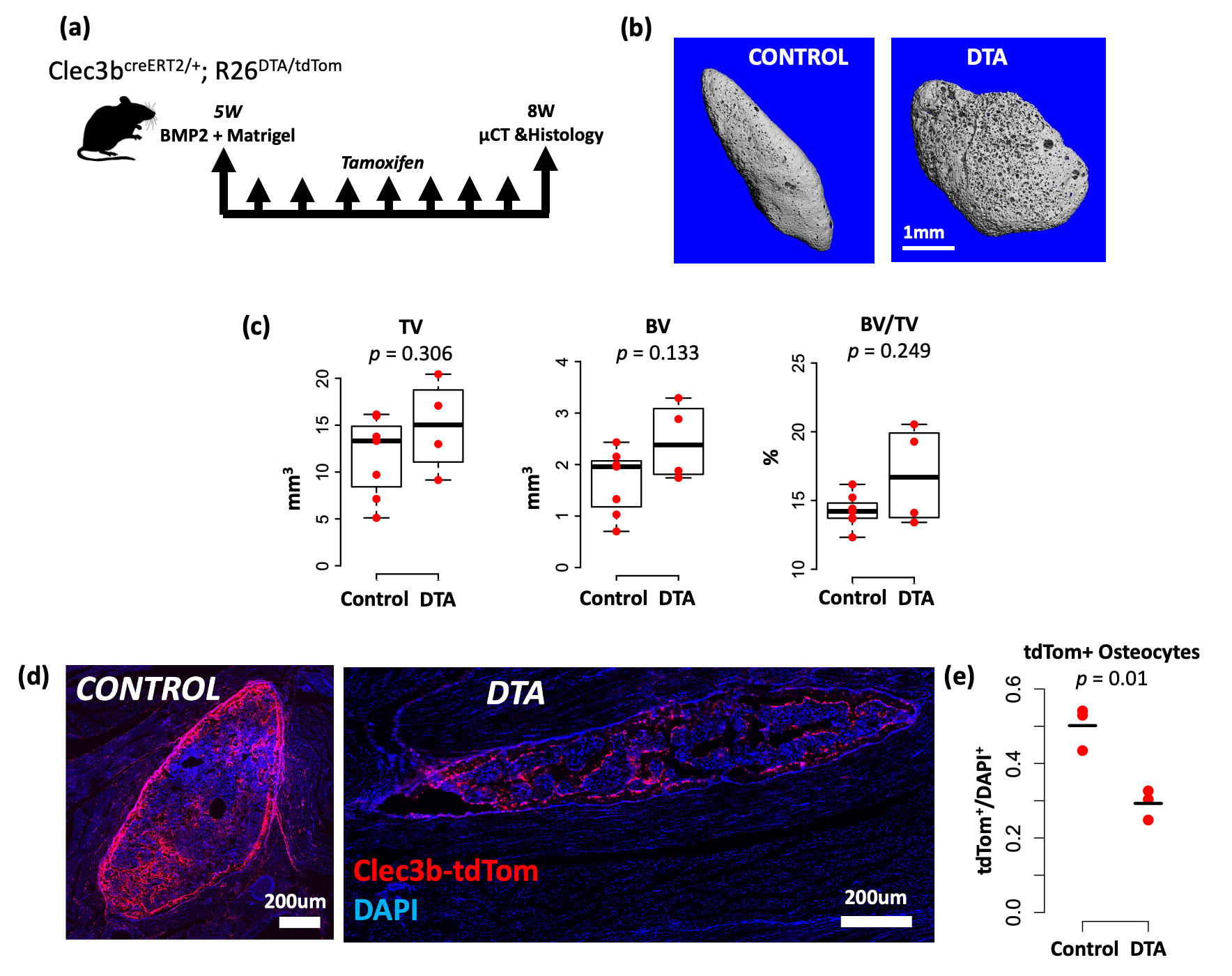


**Supplementary Figure 13: Continuous tamoxifen treatment of Clec3b^CreERT2^; R26^DTA^ mice does not reduce BMP2-induced heterotopic ossification in muscle.**

1. Clec3b^CreERT2^; R26^DTA^ mice were treated with tamoxifen every 3 days (starting the day before intramuscular BMP2 delivery) for 3 weeks.

(b-c) No reduction was observed in ectopic bones formed in the muscle.

(d) Fluorescently imaged sections of representative DTA and control specimens from Clec3b^CreERT2^; R26^DTA/tdTomato^ mice.

(e) We observed a significant reduction in tdTomato+ osteocytes in the peripheral cortex (with respect to total number of osteocytes indicated by DAPI) in DTA mice (n=3 mice/group).


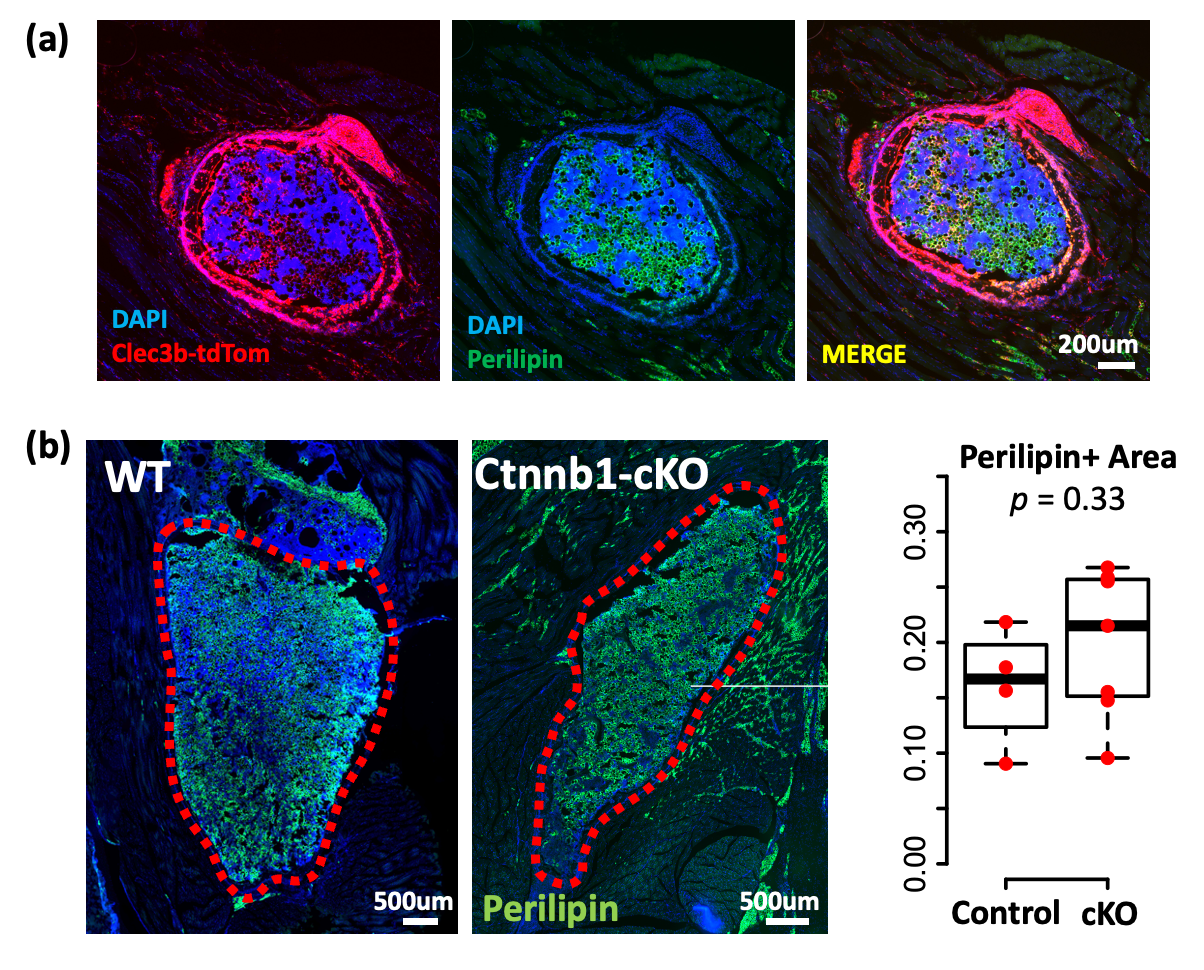


**Supplementary Figure 14: BMP2-induced ectopic bones contain perilipin+ adipocytes with Clec3b-expression history.**

**(a)** Representative images depict the presence of tdTomato+ adipocytes in BMP2-induced heterotopic ossification.

**(b)** We did not observe a change in perilipin+ cell quantity in Ctnnb1-conditional knockout mice.
